# Inhibition of the master regulator of *Listeria monocytogenes* virulence enables bacterial clearance from spacious replication vacuoles in infected macrophages

**DOI:** 10.1371/journal.ppat.1010166

**Published:** 2022-01-10

**Authors:** Thao Thanh Tran, Carmen D. Mathmann, Marcela Gatica-Andrades, Rachel F. Rollo, Melanie Oelker, Johanna K. Ljungberg, Tam T. K. Nguyen, Alina Zamoshnikova, Lalith K. Kummari, Orry J. K. Wyer, Katharine M. Irvine, Javier Melo-Bolívar, Annette Gross, Darren Brown, Jeffrey Y. W. Mak, David P. Fairlie, Karl A. Hansford, Matthew A. Cooper, Rabina Giri, Veronika Schreiber, Shannon R. Joseph, Fiona Simpson, Timothy C. Barnett, Jörgen Johansson, Wendy Dankers, James Harris, Timothy J. Wells, Ronan Kapetanovic, Matthew J. Sweet, Eleanor A. Latomanski, Hayley J. Newton, Romain J. R. Guérillot, Abderrahman Hachani, Timothy P. Stinear, Sze Ying Ong, Yogeswari Chandran, Elizabeth L. Hartland, Bostjan Kobe, Jennifer L. Stow, A. Elisabeth Sauer-Eriksson, Jakob Begun, Jessica C. Kling, Antje Blumenthal

**Affiliations:** 1 The University of Queensland Diamantina Institute, Brisbane, Australia; 2 Department of Chemistry, Umeå University, Umeå, Sweden; 3 The University of Queensland School of Chemistry and Molecular Biosciences and Australian Infectious Diseases Research Centre, Brisbane, Australia; 4 Institute for Molecular Bioscience, The University of Queensland, Brisbane, Australia; 5 ARC Centre of Excellence in Advanced Molecular Imaging, Institute for Molecular Bioscience, The University of Queensland, Brisbane, Australia; 6 Mater Research Institute – The University of Queensland, Brisbane, Australia; 7 Wesfarmers Centre for Vaccines and Infectious Diseases, Telethon Kids Institute, University of Western Australia, Nedlands, Australia; 8 Department of Molecular Biology, Umeå University, Umeå, Sweden; 9 Department of Medicine, School of Clinical Sciences at Monash Health, Faculty of Medicine, Nursing & Health Sciences, Monash University, Clayton, Australia; 10 Department of Microbiology and Immunology, University of Melbourne at the Peter Doherty Institute for Infection and Immunity, Melbourne, Australia; 11 Centre for Innate Immunity and Infectious Diseases, Hudson Institute of Medical Research and Department of Molecular and Translational Science, Monash University, Melbourne, Australia; University of Toronto, CANADA

## Abstract

A hallmark of *Listeria (L*.*) monocytogenes* pathogenesis is bacterial escape from maturing entry vacuoles, which is required for rapid bacterial replication in the host cell cytoplasm and cell-to-cell spread. The bacterial transcriptional activator PrfA controls expression of key virulence factors that enable exploitation of this intracellular niche. The transcriptional activity of PrfA within infected host cells is controlled by allosteric coactivation. Inhibitory occupation of the coactivator site has been shown to impair PrfA functions, but consequences of PrfA inhibition for *L*. *monocytogenes* infection and pathogenesis are unknown. Here we report the crystal structure of PrfA with a small molecule inhibitor occupying the coactivator site at 2.0 Å resolution. Using molecular imaging and infection studies in macrophages, we demonstrate that PrfA inhibition prevents the vacuolar escape of *L*. *monocytogenes* and enables extensive bacterial replication inside spacious vacuoles. In contrast to previously described spacious *Listeria*-containing vacuoles, which have been implicated in supporting chronic infection, PrfA inhibition facilitated progressive clearance of intracellular *L*. *monocytogenes* from spacious vacuoles through lysosomal degradation. Thus, inhibitory occupation of the PrfA coactivator site facilitates formation of a transient intravacuolar *L*. *monocytogenes* replication niche that licenses macrophages to effectively eliminate intracellular bacteria. Our findings encourage further exploration of PrfA as a potential target for antimicrobials and highlight that intra-vacuolar residence of *L*. *monocytogenes* in macrophages is not inevitably tied to bacterial persistence.

## Author summary

*Listeria monocytogenes* is a bacterial pathogen that causes severe, often fatal disease in vulnerable individuals. *Listeria monocytogenes* infects host cells, where it needs to escape from its entry vacuole to replicate inside the host cell cytoplasm and spread to neighboring cells. Inability of *Listeria* to escape from vacuoles has been associated with long-term bacterial survival inside infected immune cells and chronic infection. We discovered that inhibition of the master regulator of the bacterial factors that orchestrate the intracellular lifestyle of *Listeria monocytogenes* traps the bacteria inside their entry vacuoles and facilitates extensive bacterial replication in spacious vacuoles. Yet, the majority of infected host immune cells survive the infection and ultimately clear the bacteria. Thus, our data demonstrate that intra-vacuolar existence of *Listeria monocytogenes* in immune cells is not inevitably tied to bacterial persistence and chronic infection. We provide high resolution structural insights into how an effective inhibitory molecule interacts with the master regulator of *Listeria* virulence, which may be exploited for rational drug design.

## Introduction

The Gram-positive, facultative intracellular bacterial pathogen *Listeria (L*.*) monocytogenes* employs a suite of virulence factors for host cell invasion, escape from phagosomes (e.g. listeriolysin O, LLO; phospholipase A, PlcA), as well as subversion of host defense mechanisms and cell-to-cell spread (e.g. actin assembly-inducing protein, ActA). Expression of these *L*. *monocytogenes* virulence genes is controlled by positive regulatory factor A, PrfA [1]. Consequently, *L*. *monocytogenes* bacteria lacking PrfA expression are avirulent [2,3]. PrfA belongs to the cyclic AMP receptor protein-fumarate and nitrate reduction regulator (Crp-Fnr) family of bacterial transcription factors [4]. Transcription factors of this family bind a wide range of ligands and are activated by allosteric communication between the ligand binding site and DNA-binding helix-turn-helix (HTH) motifs. The latter are flexible in the absence of ligand [5]. PrfA is an exception in that it binds to DNA at its consensus sequence with low affinity in the absence of coactivation [5]. Affinity of PrfA for promoters is governed by PrfA-binding motifs (PrfA-box) in the DNA, and deviation from the consensus sequence reduces affinity [6]. Consequently, genes with conserved PrfA box sequences (e.g. *hly* [encodes LLO], *plcA*) are transcribed upon PrfA binding without the need for coactivation, whereas genes with lower affinity PrfA box sequences (e.g. *actA*), require allosteric coactivation of PrfA [7]. Single amino acid mutations in the PrfA coactivator binding site diminish expression of PrfA-regulated genes with either high or low affinity PrfA box sequences. However, these mutant *L*. *monocytogenes* bacteria escape their entry vacuole and translocate into the cytoplasm, which led to the conclusion that PrfA coactivation is dispensable for vacuolar escape [8].

Reduced glutathione (GSH), a low molecular weight thiol important for intracellular redox homeostasis, is thus far the only known allosteric activator of PrfA. GSH-binding to the PrfA homodimer triggers a conformational change in the HTH motifs required for efficient PrfA-DNA-interactions [9,10]. The roles for PrfA and GSH in orchestrating *L*. *monocytogenes* virulence have thus far been interrogated mainly by using genetic deletion as well as bacterial mutants with constitutively active PrfA or amino acid substitutions in the coactivator site [8,11,12]. More recently, occupation of the coactivator binding site by short peptides was shown to inhibit PrfA activity [13,14], indicating a thus far unrecognized mechanism of regulating PrfA-mediated virulence gene expression during infection. Synthetic small molecule PrfA inhibitors that occupy the coactivator binding site have been reported [15,16]. Yet, the consequences of inhibitory molecules occupying the PrfA coactivator site for *L*. *monocytogenes* pathogenesis are unknown.

Vacuolar escape is central to *L*. *monocytogenes* host subversion and pathogenesis; however, under specific circumstances *L*. *monocytogenes* can also reside in intracellular vacuoles [17]. In macrophages, a subset of intracellular bacteria exhibiting low LLO activity was found to replicate very slowly (generation time estimate 8 h) inside spacious, single-membrane *Listeria*-containing vacuoles (SLAPs) [18]. SLAPs are thought to arise from entry vacuoles through disrupted phagosome maturation during LC3-mediated phagocytosis. SLAPs are decorated with LC3 and the late endosomal/lysosomal marker LAMP1 but are non-degradative and pH neutral [17–21]. These characteristics have led to the hypothesis that SLAPs support long-term intra-macrophage persistence of *L*. *monocytogenes* in the infected host [18]. In epithelial cells, epithelial SLAP-like vacuoles (eSLAPs) were recently reported. eSLAPs share some features with SLAPs such as decoration with LC3, LAMP1, as well as neutral pH [22]. However, eSLAPs support rapid intravacuolar *L*. *monocytogenes* replication (generation time 102 min), similar to bacteria residing in the cytoplasm [22]. These entry-vacuole-derived eSLAPs are distinct from single-membrane *Listeria*-containing vacuoles (LisCVs) that were observed in long-term *in vitro* cultures of human trophoblast cells and hepatocytes [23]. LisCVs arise after several days of infection, after transitioning of bacteria through a cytosolic state, and exhibited lysosomal features (acidic pH, LAMP1^+^, cathepsin D^+^). *L*. *monocytogenes* in LisCVs were found to replicate very slowly (approx. 1% of bacteria with division septum), potentially representing a niche for long-term persistence [17,23]. Whether intravacuolar residence of *L*. *monocytogenes* in macrophages is exclusively linked to slowly replicating bacteria and long-term intracellular persistence is currently unknown.

Here, we show that inhibitory occupation of the PrfA coactivator site prevents vacuolar escape and directs *L*. *monocytogenes* into spacious intracellular vacuoles that support extensive and rapid bacterial replication in macrophages. These spacious vacuoles arise from entry vacuoles with distinct temporal dynamics of exhibiting endosomal and lysosomal features. In contrast to what is known thus far about LisCVs, the majority of infected macrophages progress to clearing the bacteria via lysosomal degradation and independent of autophagy. Our data provide unprecedented insight into the consequences of functional PrfA inhibition by targeting the PrfA coactivator site for the intracellular fate of *L*. *monocytogenes*. Importantly, our findings identify what we refer to as replication-permissive SLAPs (rSLAPs), an intra-vacuolar replication niche in macrophages that is not inevitably linked to intracellular persistence of *L*. *monocytogenes*.

## Results

### Small molecule occupation of the PrfA coactivator site suppresses *L*. *monocytogenes* virulence factor expression

**Fig 1:**
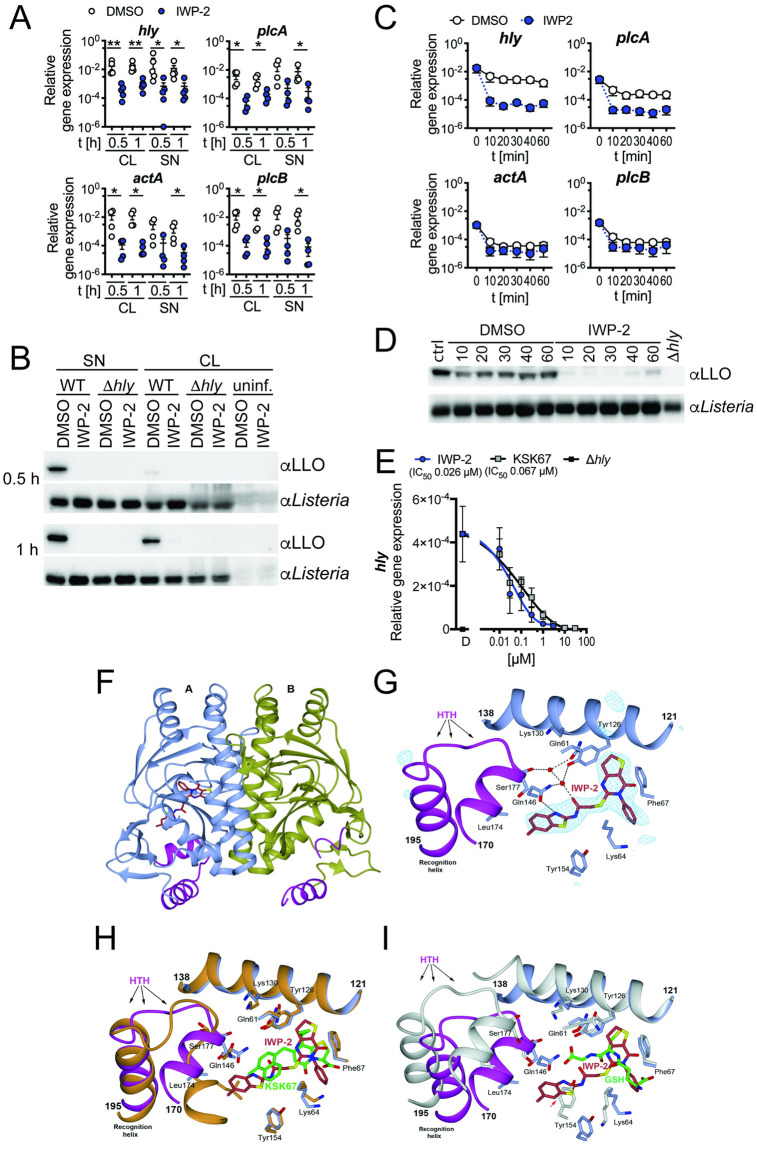
Small molecule occupation of the PrfA coactivator site impairs PrfA-driven virulence gene expression. **(A)** Significantly diminished expression of *L*. *monocytogenes* virulence genes determined by quantitative RT-PCR in cell lysates (CL) and supernatants (SN) of RAW246.7 macrophages after 0.5 and 1 h of infection in the presence of IWP-2, as compared to DMSO-treated control cells. Data are from 4–5 independent experiments; means +/- sem are indicated. Groups at each condition and time point were compared by Mann-Whitney test. **(B)** Cell lysates and supernatants of cells infected as in (A) show diminished *L*. *monocytogenes* LLO protein expression in the presence of IWP-2 compared to DMSO-treated controls, as determined by western blot. LLO-deficient Δ*hly L*. *monocytogenes* served as control. Data are representative of 3 independent experiments with similar results. **(C)** IWP-2 addition rapidly diminished PrfA-controlled *L*. *monocytogenes* virulence gene expression in brain heart infusion broth cultures grown at 37°C, as determined by qRT-PCR. Data are means +/- sem of six independent cultures analyzed across three independent experiments. **(D)** IWP-2 diminished LLO protein expression in *L*. *monocytogenes*. Bacteria were grown as in (C) and bacterial lysates analyzed by western blot at the time points indicated [min]. Untreated staring cultures (ctrl), DMSO treatment and LLO-deficient Δ*hly L*. *monocytogenes* served as controls. Data are representative of 3 independent experiments with similar results. **(E)** Dose-dependent inhibition of *L*. *monocytogenes hly* gene expression in bacterial cultures grown at 37°C, in brain heart infusion broth for 1 h in the presence of IWP-2 or the previously described PrfA inhibitor KSK67(15). DMSO [D] and LLO-deficient Δ*hly L*. *monocytogenes* served as controls. Data are means +/- sem of 4–6 independent cultures analyzed across 3–4 independent experiments. **(F)** Crystal structure of PrfA in complex with IWP-2. The PrfA homodimer with IWP-2 bound at the A_I_ site. The monomeric units A and B of PrfA are colored in blue and gold, respectively; the ligand is shown in stick representation. **(G)** Key local structural features and amino acids in proximity to IWP-2 bound at the A_I_ site. Shown is also the quality of the electron density for IWP-2. The POLDER omit electron density map in blue is calculated 5 Å around each atom of IWP-2 and contoured at 4σ. IWP-2 fits the electron density well and can be modelled into the map with high correlation coefficient [CC(1,3) = 0.85]. **(H)** Superimposition of the N-terminal domains (residues 2–121) of PrfA structures harboring the 2-pyridone compound KSK67 (PDB ID 6EUT) and IWP-2. Both the N-terminal domain and the C-terminal domain (residues 138–237, which includes the DNA binding HTH motif) superimpose well. The PrfA-2-pyridone structure is shown in orange with KSK67 shown in green stick representation. **(I)** Superimposition of the N-terminal domains of GSH- (PDB ID 5LRR) and IWP-2-bound PrfA structures. The PrfA:GSH structure is shown in white with GSH shown in green stick representation. Due to the collapsed N-terminal domain in GSH-activated PrfA only parts of the N-terminal domain superimpose. Notably, Tyr154 in GSH-activated PrfA is positioned at the same position as the methyl-benzothiazole group of IWP-2 (red arrow). As a result, in the IWP-2-bound PrfA structure, even though it is folded, the HTH motif has a position not compatible with DNA binding.

We investigated the impact of a series of small molecule inhibitors that target mammalian cell signaling cascades on *L*. *monocytogenes* control by infected macrophages. Through these analyses, we discovered that inhibitor of WNT production, IWP-2 (N-(6-methyl-1,3-benzothiazol-2-yl)-2-[(4-oxo-3-phenyl-6,7-dihydrothieno[3,2-d]pyrimidin-2-yl)sulfanyl]acetamide; C_22_H_18_N_4_O_2_S_3_), significantly impaired expression of PrfA-controlled *L*. *monocytogenes* virulence genes (e.g. *hly*, *plcA*, *plcB*, *actA*) [24] (Fig 1A and 1B and S1A Fig). Expression of *prfA* showed a similar trend but was less affected by IWP-2 (S1B Fig), indicating dominance of other regulators, such as sigma B [25–27], governing *prfA* expression prior to and early during encounter with macrophages. Expression of PrfA-independent genes (e.g. *flaA*, *murA*, *pfkA*) was not affected by IWP-2 (S1B Fig).

PrfA-regulated virulence factors are weakly expressed outside the mammalian host but greatly induced at temperatures >30°C (e.g. upon ingestion) through a structural change in the untranslated leader RNA of PrfA [28]. The promoter region governing *hly* and *plcA* expression, both genes critical for early phagosomal escape, has high-affinity for PrfA, whereas virulence genes required in subsequent steps of host cell infection, such as *actA* and *plcB*, have lower affinity PrfA-box motifs and require allosteric PrfA coactivation [1]. Thus, we next questioned whether IWP-2 directly affected *L*. *monocytogenes* virulence factor expression, in the absence of host cells. IWP-2 rapidly diminished expression of *hly* and *plcA* mRNA, as well as LLO protein in *L*. *monocytogenes* cultured in liquid medium at 37°C in the absence of host cells, with only minor impact on *actA* and *plcB* and expression (Fig 1C and 1D). IWP-2 was 2.6-times more potent in inhibiting *hly* expression of *L*. *monocytogenes* in liquid culture compared to the recently described bicyclic 4-(1-naphthylmethyl)-2-pyridone PrfA inhibitor, KSK67 (15); IWP-2 IC_50_ = 0.026 μM versus KSK67 IC_50_ = 0.067 μM (Fig 1E). IWP-2 was originally described as inhibitor of the mammalian acyltransferase Porcupine, PORCN [29]. We therefore assessed whether a structurally unrelated PORCN inhibitor had similar effects and found that the PORCN inhibitor LGK-974 did not impair *L*. *monocytogenes* virulence gene expression (S1C and S1D Fig).

Based on these observations, we hypothesized that IWP-2 is a PrfA inhibitor. To address this, we initially performed docking studies to identify potential binding sites of IWP-2 within PrfA based on docking scores. We compared the docking scores of IWP-2 to those of previously described 2-pyridone-based inhibitors [16], as well as the inactive LGK-974, to identify potential interactions that could explain effective inhibition of PrfA by IWP-2. The GSH coactivator binding site is also referred to as the tunnel site [5], or the A_I_ and B_I_ sites in PrfA monomers A and B, respectively [15]. The docking of IWP-2, but not LGK-974, revealed docking scores at the A_I_ site similar to those reported for cyclic 2-pyridones (S1 Methods, S2A–S2F Fig). Collectively, the docking analyses suggested that IWP-2 may inhibit PrfA functions by directly binding the A_I_ pocket of the PrfA homodimer, similar to PrfA-inhibitory cyclic 2-pyridones [16]. To verify and elucidate structural details of PrfA inhibition by IWP-2, we determined the crystal structure of the PrfA:IWP-2 complex at 2.0 Å resolution (S1 Table). The asymmetric unit of the PrfA:IWP-2 complex crystals contained one PrfA dimer composed of monomers A and B. Difference Fourier and Polder electron density maps unambiguously confirmed binding of IWP-2 to PrfA in monomer A, at the A_I_ site, but not the B_I_ site in monomer B (Fig 1F). IWP-2 bound at the A_I_ site with mostly hydrophobic interactions, as well as one direct hydrogen bond with the side chain of Gln146, and one water-mediated hydrogen with the side chains of Gln61, Tyr126 and Gln146 (Fig 1G). The ring-fused pyrimidone group of IWP-2 was positioned at site S1 [16] in the vicinity of the aromatic side chains of residues Tyr126 and Phe67 (Fig 1G). Overall, with the exception of Gln146, the amino acid residues that are part of the A_I_ site displayed similar conformations in 2-pyridone KSK67- and IWP-2-bound PrfA (Fig 1H) [15,16]. The methyl-benzothiazole group of IWP-2 was directed towards, and pushed on, the first helix of the HTH motif. This resulted in a shift of the helix and folding into a structural conformation similar to that present in the GSH-activated PrfA structure (Fig 1I) [10]. However, the binding of IWP-2 likely prevents the collapse of the N-terminal PrfA domain, a process otherwise necessary to line up the folded HTH motifs—one in each monomer—in positions compatible with palindromic DNA binding (Fig 1I) [10]. In the PrfA monomer B, where no IWP-2 bound, the HTH motif was not folded (Fig 1F).

Taken together, our functional and structural data identified IWP-2 as a PrfA inhibitor that occupies the coactivator site.

### PrfA inhibition delays intracellular replication of *L*. *monocytogenes*

**Fig 2:**
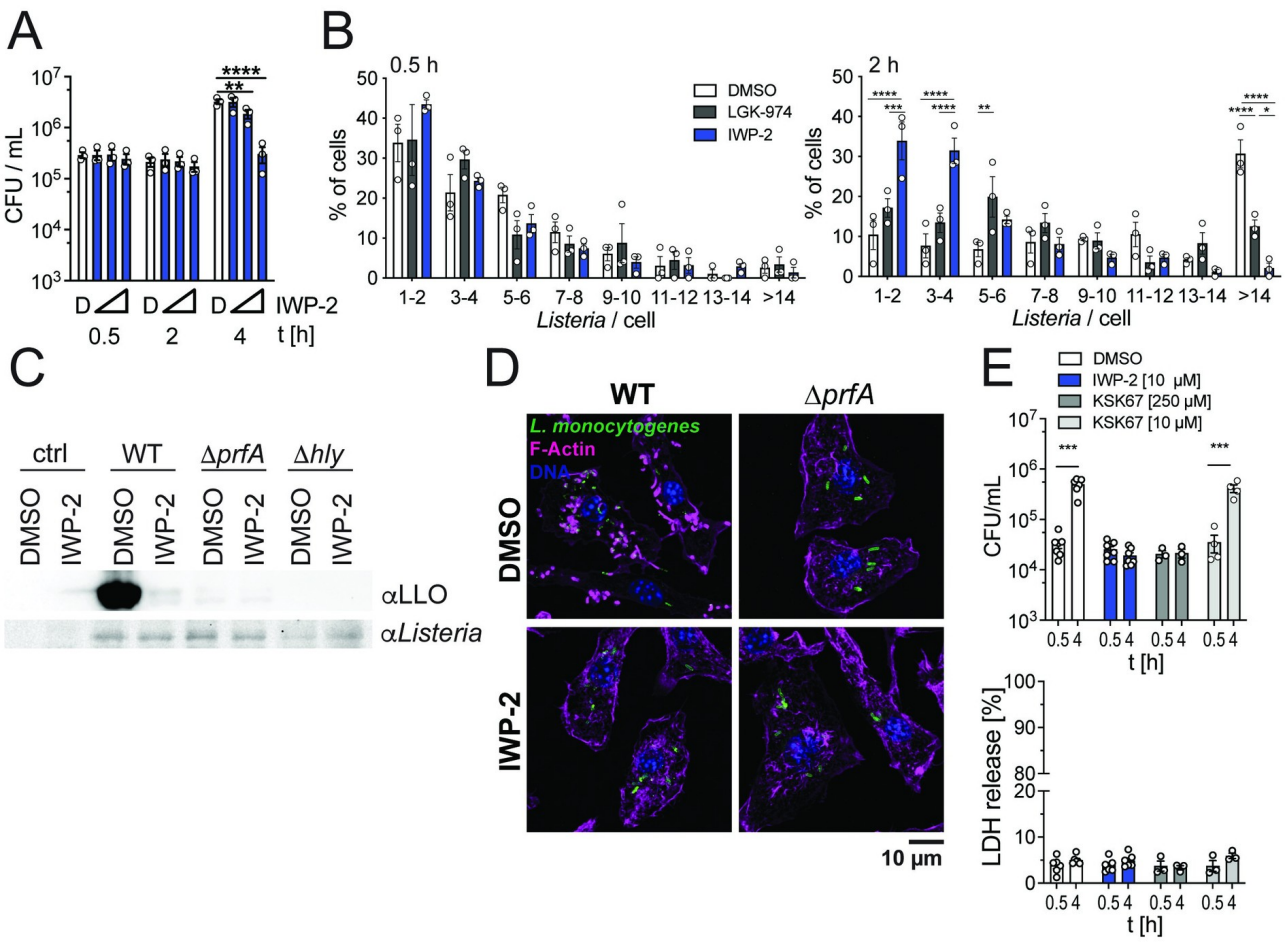
PrfA inhibition limits early *L*. *monocytogenes* replication in infected host cells. **(A)** IWP-2 (0.1, 1, 10 μM) dose-dependently diminished intracellular *L*. *monocytogenes* burden at 2–4 h post-infection of murine bone marrow-derived macrophages (BMM). D = DMSO solvent control. Data points represent 3 independent experiments, each performed in technical triplicates; means +/- sem are indicated. Two-way ANOVA with Dunnett multiple comparison correction. **p<0.01, ****p<0.0001 **(B)** Quantification of GFP-expressing *L*. *monocytogenes* harbored by BMM shows that IWP-2 (10 μM) did not affect the ability of *L*. *monocytogenes* to infect macrophages (t = 0.5 h) but limited early intracellular *L*. *monocytogenes* replication (t = 2 h). DMSO and the PORCN inhibitor LGK-974 (10 μM) served as controls. Graphed data are means +/- sem of >150 cells analyzed by fluorescence microscopy in 3 independent experiments. Two-way ANOVA with Tukey multiple comparison correction. (**C**) Western blot analyses show that IWP-2 (10 μM) diminished expression of LLO in BMM infected with wild type *L*. *monocytogenes* (4 h post-infection). Macrophages infected with Δ*prfA* and Δ*hly* showed no detectable LLO expression; uninfected BMM served as additional control (ctrl). (**D**) Lack of actin tail formation in BMM infected with wild type *L*. *monocytogenes* in the presence of IWP-2, similar to infection with Δ*prfA* mutant bacteria (4 h post-infection). Immune fluorescence microscopy images representative of at least 3 independent experiments are shown. Scale bar 10 μm. **(E)** PrfA inhibitor IWP-2 more potently inhibited early intracellular *L*. *monocytogenes* replication in BMM than KSK67, as determined by assessing viable intracellular bacteria at 4 h post-infection. Equivalent host cell viability was confirmed by LDH release. Notably, neither IWP-2 nor KSK67 impaired the ability of *L*. *monocytogenes* to infect BMM (t = 0.5 h). Data points represent 3–7 independent individual experiments, each performed in technical triplicates; means +/- sem are indicated. Groups were compared by unpaired t-test.

Our data indicated that inhibitor occupation of the PrfA coactivator site reduced virulence gene expression across the PrfA regulon (Fig 1). This did not affect the ability of *L*. *monocytogenes* to infect macrophages (Fig 2A and 2B and S3A Fig) and non-phagocytic epithelial cells (S3B Fig). However, within 2–4 hours of infection, PrfA inhibition prevented early replication of intracellular *L*. *monocytogenes* when compared to control-treated cells (Fig 2A and 2B and S3A and S3B Fig). This was accompanied by almost complete lack of detectable LLO expression in infected host cells and lack of polymerized actin clouds and tails associated with intracellular bacteria, phenotypes similar to *prfA*-deficient *L*. *monocytogenes* (Fig 2C and 2D). IWP-2 was substantially more potent in restricting early intracellular replication when compared to the 2-pyridone KSK67 (Fig 2E), consistent with higher potency of IWP-2 in inhibiting PrfA-controlled gene expression (Fig 1E). Neither PrfA inhibitor affected host cell viability (Fig 2E).

We noted that addition of IWP-2 after initial infection of macrophages by *L*. *monocytogenes* also effectively reduced intracellular bacterial burden (S3C Fig), suggesting that IWP-2 can target PrfA within infected cells. We confirmed that the PrfA inhibitor did not impair *L*. *monocytogenes* replication in broth cultures *in vitro* (S3D Fig). IWP-2 was originally identified as an inhibitor of the mammalian acyltransferase PORCN [29], yet experiments using siRNA-mediated knock-down as well as the PORCN inhibitor LGK-974 established that the IWP-2 impact on intracellular *L*. *monocytogenes* burden was likely independent of PORCN activity (S3E and S3F Fig). We further observed that *L*. *monocytogenes* displayed a unique susceptibility to IWP-2 when compared to Gram-positive and Gram-negative bacterial pathogens that do not express PrfA (S3G Fig).

**Fig 3:**
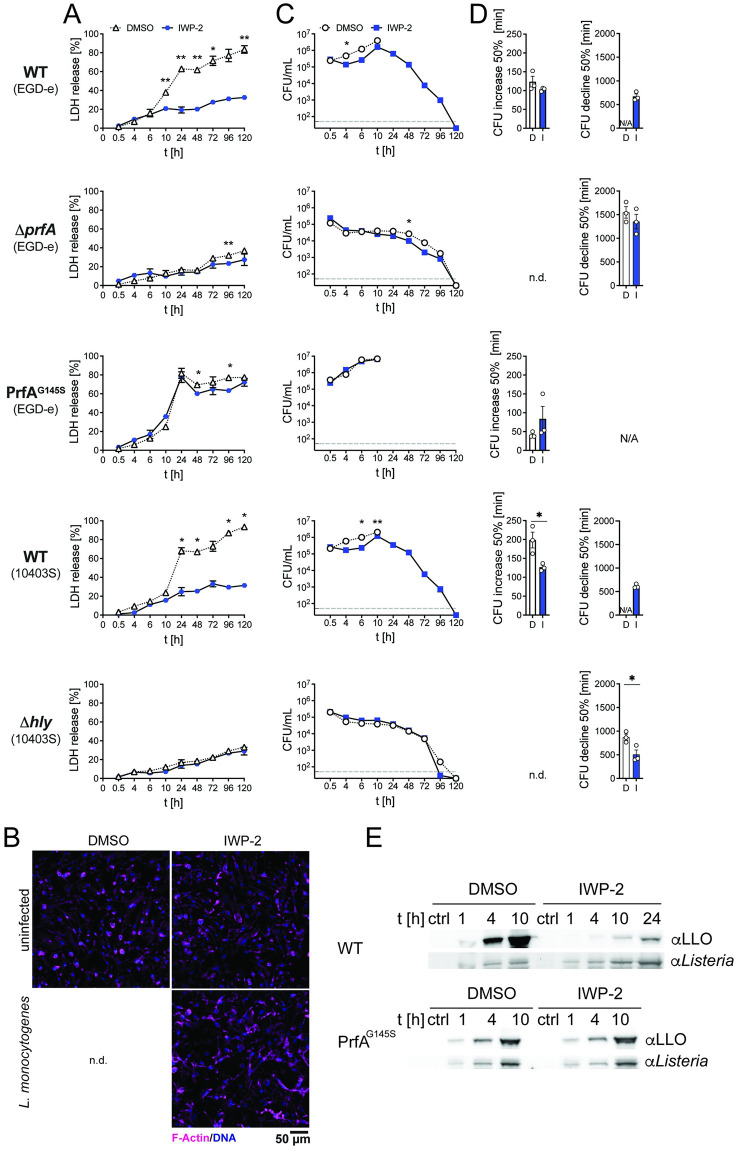
PrfA inhibition facilitates survival of infected macrophages alongside rapid expansion but ultimate elimination of intracellular *L*. *monocytogenes*. **(A)** Survival of BMM determined by LDH release assay upon infection with wild type (WT) *L*. *monocytogenes* EGD-e and 10403S, Δ*prfA*, PrfA^G145S^, and Δ*hly* in the presence of PrfA inhibitor IWP-2 (10 μM) or DMSO (solvent control) at the indicated time points post-infection. Data are means +/- sem of 3 independent experiments, each performed in technical triplicates. Two-way ANOVA, Sidak multiple comparison test; *p<0.05, **p<0.01. **(B)** Immune fluorescence images of BMM infected with wild type *L*. *monocytogenes* or left uninfected, in the presence of PrfA inhibitor IWP-2 (10 μM) or DMSO at 5 days post-infection. No imageable cells remained in cultures infected in the presence of DMSO. Data are representative of at least three independent experiments. **(C)** Intracellular bacterial burden upon infection with wild type (WT) *L*. *monocytogenes* EGD-e and 10403S, Δ*prfA*, PrfA^G145S^, and Δ*hly* in the presence of PrfA inhibitor IWP-2 (10 μM) or DMSO (solvent control) at the indicated time points post-infection. Data are means +/- sem of 3 independent experiments, each performed in technical triplicates. **(D)** For the data in (C), the time required for a 50% increase in CFU (WT *L*. *monocytogenes* EGD-e, 10403S 4–10 h post-infection; PrfA^G145S^ 4–6 h post-infection) and 50% decline in CFU (10–96 h post-infection) of *L*. *monocytogenes* in infected macrophage cultures in the presence of DMSO (D) and IWP-2 (I) were calculated. Data are means +/- sem of 3 independent experiments, each performed in technical triplicates. N/A not applicable; n.d. not determined. Unpaired two-tailed *t-test*, *p<0.05. **(E)** LLO expression in BMM infected with WT *L*. *monocytogenes*, Δ*prfA*, PrfA^G145S^, and Δ*hly* in the presence of DMSO or IWP-2 (10 μM) for the indicated times. Untreated starting cultures served as control (ctrl). Western blot representative of 2 independent experiments.

Infection with *L*. *monocytogenes* triggers macrophage death (Fig 3A). However, in the presence of PrfA inhibitor, limited cell death occurred in macrophage cultures infected with wild type *L*. *monocytogenes*, reminiscent of macrophage survival upon infection with *L*. *monocytogenes* deficient in PrfA or LLO expression (Fig 3A and 3B). This allowed us to extend the analyses on the impact of PrfA inhibition on intracellular bacterial burden in macrophages. After the aforementioned initial delay of intracellular replication of wild type *L*. *monocytogenes* in the presence of PrfA inhibitor, we observed a sharp increase in intracellular viable bacteria between 4 and 10 hours after infection (Fig 3C). This resulted in intracellular bacterial burden approaching that of DMSO-treated cultures by 10 hours post-infection (ratio CFU in DMSO/IWP-2 between 1.2–3.3) (Fig 3C). Over this period, the rate with which viable intracellular *L*. *monocytogenes* increased in macrophage cultures in the presence of PrfA inhibitor was elevated compared to DMSO-exposed control cultures (*L*. *monocytogenes* EGD-e: DMSO 123.5 +/- 14.6 min, IWP-2 102.9 +/- 2.8 min; average difference 17%; *L*. *monocytogenes* 10403S: DMSO 198.5 +/- 21 min; IWP-2 126.5 +/- 5.5 min, average difference 36%) (Fig 3D). This was accompanied by markedly reduced LLO expression (Fig 3E). It is important to note that this increase in CFU likely reflects a composite of *L*. *monocytogenes* replication, but also killing of intracellular bacteria by macrophages as well as gentamycin-mediated killing of bacteria released from dying cells into the culture medium. In contrast to wild type *L*. *monocytogenes*, IWP-2 did not delay bacterial replication or limit macrophage death of macrophages infected with *L*. *monocytogenes* expressing the constitutively active PrfA^G145S^, and IWP-2 did not affect bacterial uptake or lack of intracellular replication of Δ*prfA* and Δ*hly L*. *monocytogenes* (Fig 3A–3D). These observations further support the notion that IWP-2 binding in the PrfA coactivator site inhibits *L*. *monocytogenes* virulence factor expression and initial intracellular bacterial replication. Collectively, these data show that PrfA inhibition delays, but does not prevent extensive intracellular *L*. *monocytogenes* replication, accompanied by significant protection of macrophages from death.

### PrfA inhibition enables *L*. *monocytogenes* replication in spacious vacuoles

**Fig 4:**
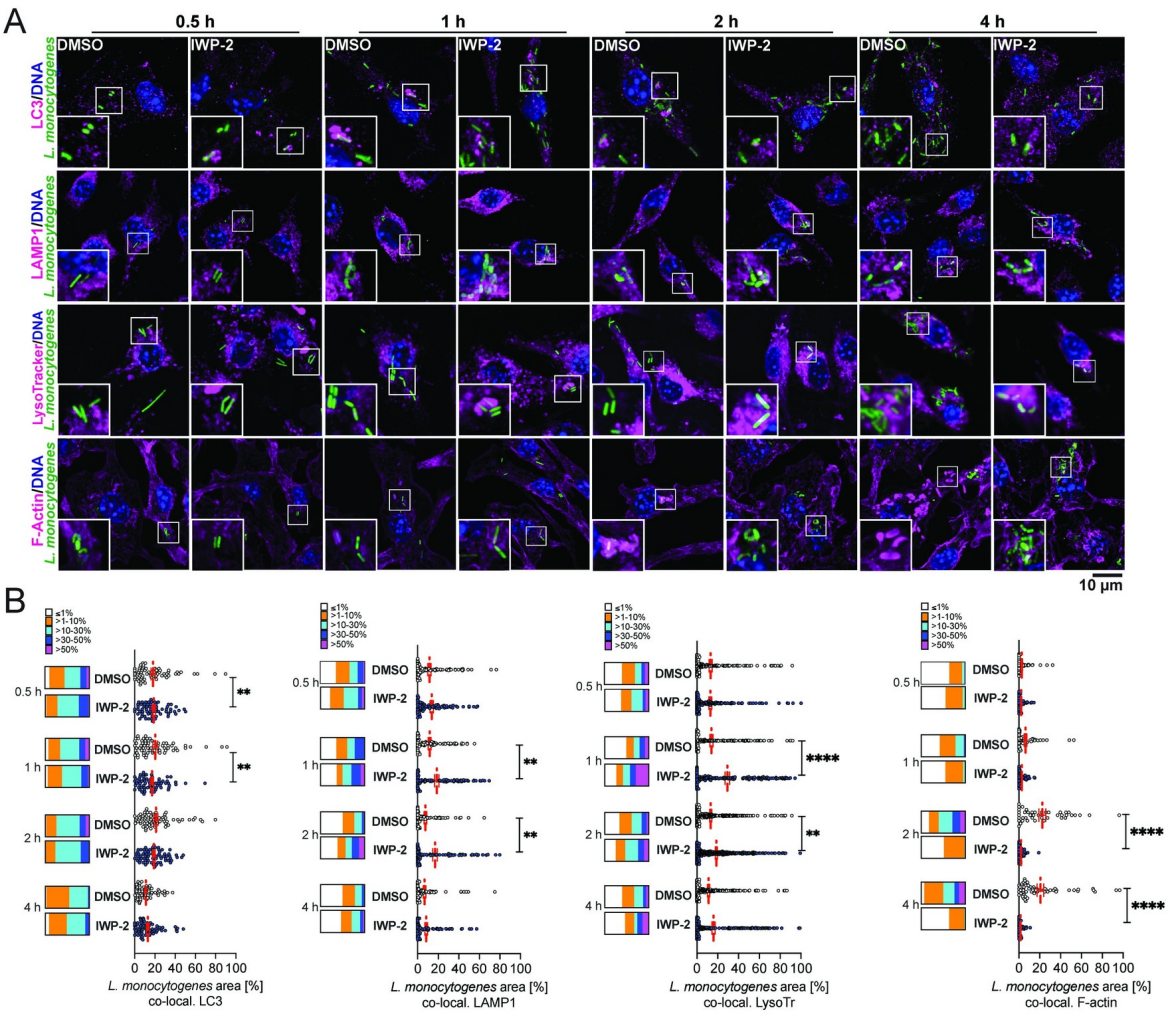
Delayed early intracellular bacterial replication upon PrfA inhibition is associated with transition of *L*. *monocytogenes* through acidified late endosomal compartments and lack of actin polymerization. **(A)** Confocal fluorescence microscopy of murine bonemarrow-derived macrophages (BMM) infected with wild type *L*. *monocytogenes* for the indicated time points and co-stained for LC3, LAMP-1, acidified compartments (LysoTracker), and filamentous (F-) actin (phalloidin). Images are representative of at least 6 independent experiments (scale bar 10 μm). **(B)** Quantitative analyses of *L*. *monocytogenes* co-localization in each cell with individual cellular markers. Scatter plots show data points from 83–108 cells per condition and time point, analyzed cumulatively across 3 independent experiments. Means +/- sem are indicated. Box plots are binned data: ≤1%, >1–10%, >10–30%, >30–50%, >50% of *L*. *monocytogenes* signal per cell co-localized with cellular marker. Comparisons between DMSO and IWP-2 by 2-way ANOVA with Tukey correction for multiple comparisons; **p<0.01, ****p<0.0001.

To better understand the cellular events aligned with the initial delay and subsequent extensive intracellular bacterial replication upon PrfA inhibition, we assessed the spatial and temporal interaction of *L*. *monocytogenes* with endosomal and cytosolic compartments. At 1 and 2 hours post-infection, in the presence of IWP-2, we observed elevated *L*. *monocytogenes* association with the late endosomal/lysosomal marker LAMP1^+^ (20) and acidified compartments, with minor differences in the extent to which *L*. *monocytogenes* associated with LC3 (Fig 4A and 4B). These observations suggest that PrfA inhibition enhances *L*. *monocytogenes* association with acidified late endosomal compartments coinciding with the delay in early intracellular bacterial replication. However, the subsequent increase of viable intracellular *L*. *monocytogenes* from 4 hours onwards (Fig 3C) was preceded by a marked decline of LAMP1 and lysotracker co-localization with *L*. *monocytogenes* (Fig 4B).

**Fig 5:**
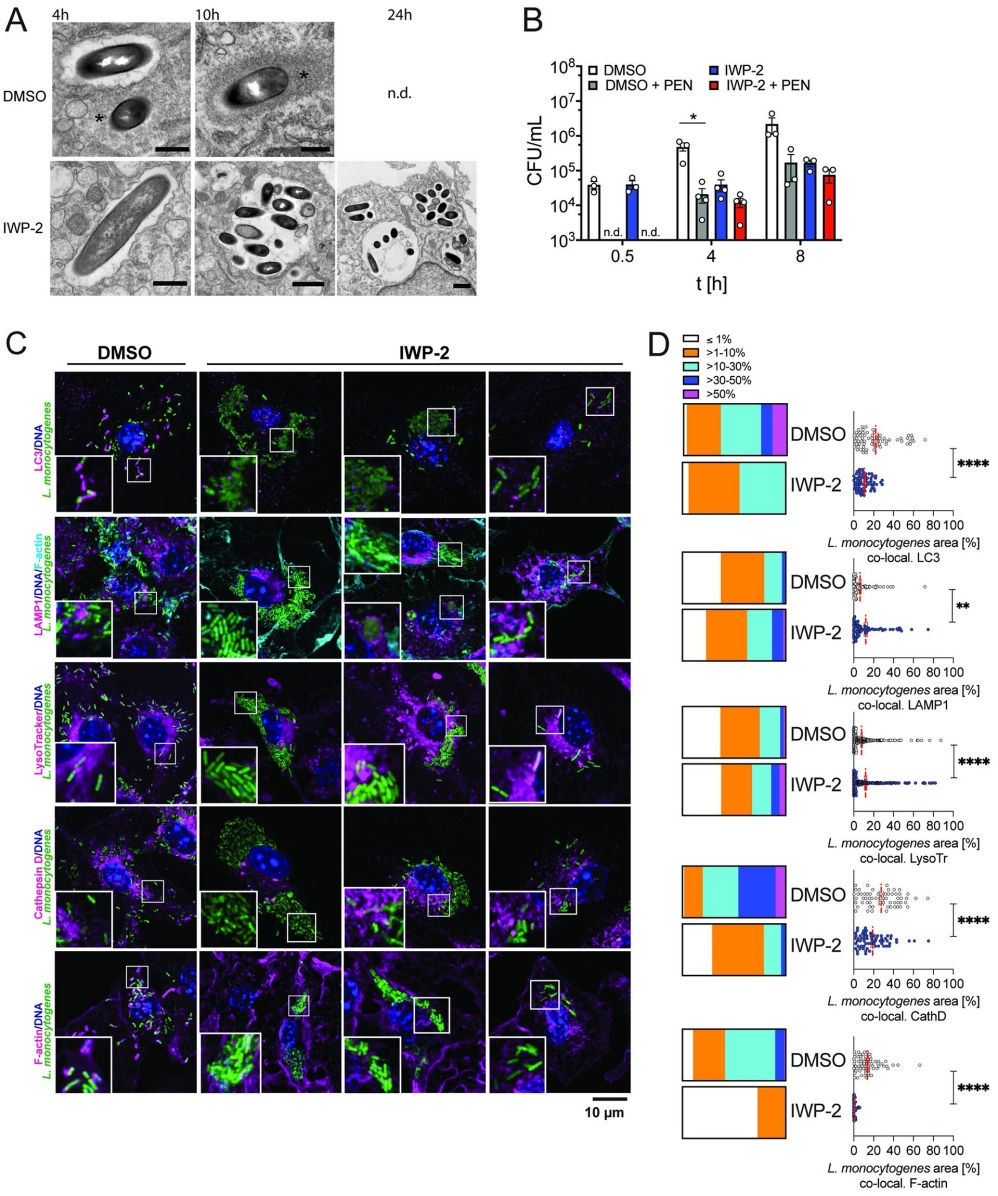
PrfA inhibition enables extensive intra-macrophage replication of *L*. *monocytogenes* inside spacious vacuoles. **(A)** *L*. *monocytogenes* in IWP-2-treated mouse bone marrow-derived macrophages (BMM) reside and replicate inside spacious single-membrane vacuoles as revealed by transmission electron microscopy (TEM) (scale bars 0.5 μm DMSO 4 h, 10 h, IWP-2 4 h; 1 μm IWP-2 10 h, 24 h; asterisks indicate polymerized actin associated with cytoplasmic bacteria). Similar observations were made in two independent experiments (n.d., not determined). **(B)** *L*. *monocytogenes* replicating inside spacious vacuoles are protected from penicillin. BMM were infected with *L*. *monocytogenes* in the presence of IWP-2 (10 μM) or DMSO as control. Protection from penicillin (60 μg/ml) addition to infected cells was determined by assessing intracellular bacterial burden at the indicated time points. Data are means +/- sem of 3–4 independent experiments, each performed in triplicates. Two-way ANOVA with Tukey correction for multiple comparisons; * P<0.05. **(C,D)** Spacious vacuoles that support extensive *L*. *monocytogenes* replication are not decorated with LC3 and LAMP1, and are non-degradative. (C) Maximum projection spinning-disk confocal microscopy of BMM infected with *L*. *monocytogenes* for 8 h in the presence of IWP-2 (10 μM) or DMSO analyzed for co-localization with LC3, LAMP1, acidification (LysoTracker), lysosomal protease cathepsin D, and filamentous (F)-actin (scale bar 10 μm). Representative images of at least 3 independent experiments performed for each condition. (D) Co-localization of total *L*. *monocytogenes* signal per cell with signal for each cellular marker. Scatter plots are data points from 83–108 cells per condition analyzed cumulatively across 3 independent experiments; means +/- sem are shown. Box plots are binned data: ≤1%, >1–10%, >10–30%, >30–50%, >50% of *L*. *monocytogenes* signal per cell co-localized with cellular marker. Mann-Whitney test; **p<0.01, ****p<0.0001.

**Fig 6:**
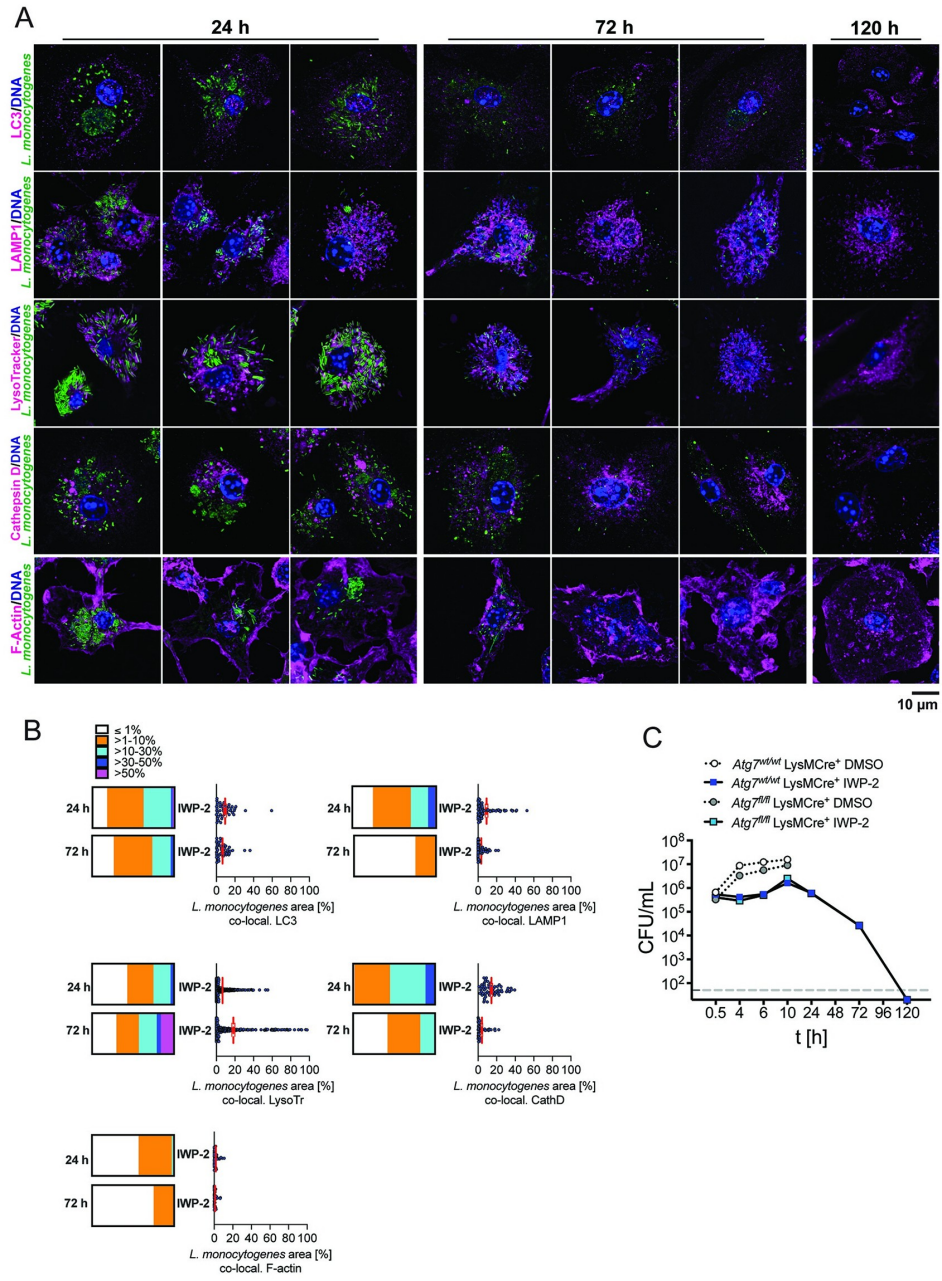
Progressive elimination of *L*. *monocytogenes* from spacious replication vacuoles in infected macrophages is associated with acquisition of lysosomal features independent of autophagy. **(A)** Spinning-disk confocal fluorescence microscopy of mouse bone marrow-derived macrophages (BMM) infected with *L*. *monocytogenes* in the presence of IWP-2 (10 μM) at 24, 72, 120 h post-infection. Cells were co-stained for LC3, LAMP1, acidified (LysoTracker) and cathepsin D-positive, lysosomal compartments, and filamentous (F-) actin (scale bar 10 μm). Images are representative of at least 6 independent experiments. **(B)** Co-localization of total *L*. *monocytogenes* signal per cell with signal for each cellular marker. Scatter plots are data points from 83–108 cells per condition analyzed cumulatively across 3 independent experiments; means +/- sem. Box plots are binned data: ≤1%, >1–10%, >10–30%, >30–50%, >50% of *L*. *monocytogenes* signal per cell co-localized with cellular marker at 24 and 72 h post-infection. No co-localization at 120 h due to lack of detectable intracellular *L*. *monocytogenes*. **(C)** Intracellular *L*. *monocytogenes* burden in IWP-2 and DMSO-treated *Atg7* deficient (*Atg7*^fl/fl^ LysMCre^+^) and matched control (*Atg7*^wt/wt^ LysMCre^+^) BMM at the indicated time points. Data are means +/- sem of 3 independent experiments each performed in triplicates.

Presence of the PrfA inhibitor resulted in a lack of polymerized actin clouds and tails (Fig 4A and 4B), which are a hallmark of cytosolic intracellular *L*. *monocytogenes*. One possible explanation is that this reflects effective inhibition of PrfA-driven expression of ActA (Fig 1A), which is required for actin polymerization [30]. In addition, reduced LLO expression (Figs 1B, 2C and 3E) and initial association of *L*. *monocytogenes* with late endosomal compartments (Fig 4A and 4B) led us to hypothesize that PrfA inhibition resulted in retention of *L*. *monocytogenes* in vacuolar compartments. Indeed, *L*. *monocytogenes* in IWP-2-treated macrophages remained inside single-membrane vacuoles by 4 hours post-infection (Fig 5A). In contrast, both cytoplasmic and intravacuolar *L*. *monocytogenes* occurred in DMSO-treated control cells (Fig 5A). At the time of the steep rise in viable intracellular bacteria between 4 and 10 hours post-infection (Fig 3C), IWP-2-treated macrophages harbored numerous *L*. *monocytogenes* inside spacious vacuoles (Fig 5A). Intracellular *L*. *monocytogenes* replicating in the presence of the PrfA inhibitor were protected from penicillin, which effectively kills bacteria replicating in the cytoplasm (Fig 5B). Together with enhanced survival of infected macrophages (Fig 3A and 3B), which usually die via pyroptosis upon sensing of intracytoplasmic *L*. *monocytogenes* [31], these data suggest that PrfA inhibition enables replication of *L*. *monocytogenes* inside spacious vacuoles, shielded by intact membranes. Bacterial LLO, but not ActA, was required for *L*. *monocytogenes* replication upon PrfA inhibition (Fig 4C and S4 Fig), suggesting that these spacious vacuoles arise from entry vacuoles and do not require re-infection of neighboring cells as described for LisCVs [23]. As these vacuoles shared features of SLAPs [18], but supported extensive intravacuolar *L*. *monocytogenes* replication as reported for eSLAPs [22], we named them replication-permissive SLAPs (rSLAPs). At the time of high intra-vacuolar *L*. *monocytogenes* burden, neither LC3 nor LAMP1 decorated the perimeter of rSLAPs, and the spacious vacuoles were not acidified and largely void of the lysosomal endo-protease cathepsin D (Fig 5C and 5D). Nevertheless, it is noteworthy that on occasion, single bacteria or small bacterial aggregates were observed, either alongside rSLAPs within the same cell or in cells without rSLAPs. In these cases, co-localization of *L*. *monocytogenes* with LAMP1, cathepsin D and acidified compartments did occur and in rare instances bacterial aggregates were surrounded by a perimeter of LAMP1 (Fig 5C and 5D). These structures may represent SLAPs. Yet lysotracker-positive bacteria and the dim fluorescence signal of GFP-expressing *L*. *monocytogenes* suggests that these bacteria were contained in acidified compartments (quenched GFP signal). Thus, these bacteria might represent early examples of bacterial delivery into lysosomal compartments destined for degradation as discussed below (Fig 6). We further noticed close spatial proximity of mitochondria to rSLAPs (S5A and S5B Fig). While there are reports that intravacuolar bacteria manipulate mitochondrial function to support intracellular replication [32], we found no evidence that mitochondrial function was required for intra-vacuolar *L*. *monocytogenes* replication (S5C Fig). Consistent with intravacuolar localization and/or effective PrfA inhibition, the large intracellular aggregates of *L*. *monocytogenes* in PrfA-inhibitor-treated macrophages remained devoid of actin tails (Fig 5C and 5D). This contrasted the actin tails associated with *L*. *monocytogenes* in DMSO-treated control macrophages, which at this stage of infection also attracted LC3 (Fig 5C and 5D).

In summary, these data demonstrate that PrfA inhibition restricts *L*. *monocytogenes* to spacious vacuoles in macrophages that are permissive for rapid and extensive bacterial replication. These rSLAPS share features with SLAPs: (i) they likely arise from entry vacuoles in the context of low LLO expression, (ii) are likely derived from a late endosomal compartment, and (iii) are non-degradative at the time of extensive bacterial replication [17–21]. In contrast to SLAPs, rSLAPs are permissive for extensive *L*. *monocytogenes* replication in cultured macrophages over a period of several hours, and are no longer decorated with LAMP1 and LC3 as bacterial replication progresses.

### PrfA inhibition licenses macrophages to clear high intracellular burden of *L*. *monocytogenes*

Slow intravacuolar replication in macrophages (SLAPs) and epithelial cells (LisCVs) has been implicated in long-term intracellular persistence of *L*. *monocytogenes* [18,23]. In contrast, our extended infection experiments showed that the sharp rise in intracellular viable *L*. *monocytogenes* was followed by progressive decline until no viable bacteria were recovered by 5 days post-infection (Fig 3C). The rate by which intracellular burden of viable *L*. *monocytogenes* declined upon PrfA inhibition between 10–120 h post-infection was accelerated, compared to macrophage clearance of Δ*prfA* and Δ*hly* mutant *L*. *monocytogenes* (wild type *L*. *monocytogenes* EGD-e 619.8 +/- 20.5 min; Δ*prfA L*. *monocytogenes* EGD-e (DMSO) 1545.9 +/- 121.9 min; wild type *L*. *monocytogenes* 10403S: 613.8 +/- 25.3 min, Δ*hly L*. *monocytogenes* (DMSO) 10403S 873.1 +/- 66.6 min) (Fig 3C and 3D). Similarly, the number of viable intracellular Δ*hly L*. *monocytogenes* declined more rapidly in the presence of IWP-2 (505.4 +/- 97.3 min), compared to DMSO treated cells (Fig 3C and 3D). In contrast, the PrfA inhibitor did not accelerate macrophage clearance of the Δ*prfA* mutant (DMSO: 1545.9 +/- 121.9 min; IWP-2: 1352.4 +/- 154.1 min) (Fig 3C and 3D), further affirming specificity of the effect exerted by IWP-2.

The progressive decline of intracellular wild type *L*. *monocytogenes* was accompanied by increasing acidification of bacteria-containing compartments, enclosure of bacteria in smaller LAMP1^+^ compartments, and co-localization with cathepsin D (Fig 6A and 6B), commensurate with lysosomal delivery of *L*. *monocytogenes*. LC3 did not decorate *Listeria*-containing vacuoles during these events (Fig 6A), and neither bacterial replication nor clearance upon PrfA inhibition were altered in *Atg7*-deficient macrophages (Fig 6C). These observations suggested that lysosomal delivery of *L*. *monocytogenes* was not driven by autophagy. Importantly, and in contrast to bacterial persistence associated with LisCVs [23] and SLAPs [18], *L*. *monocytogenes* was progressively eliminated from macrophages and without killing the majority of infected host cells (Figs 3A–3C and 6A). Collectively, these data show that PrfA inhibition licenses macrophages to survive *L*. *monocytogenes* infection and progressively clear a high load of intravacuolar bacteria through lysosomal degradation.

## Discussion

*L*. *monocytogenes* is equipped with a suite of virulence factors that are geared towards escape from host vacuoles to enable cytoplasmic replication of the bacteria and infection of neighboring cells. Yet, emerging evidence shows that this prototypical intracytoplasmic pathogen can occupy distinct intracellular vacuoles. Thus, it is timely to define how *L*. *monocytogenes* utilizes the host cell niche, including intracellular vacuoles. In this study, we discovered that inhibition of PrfA activity prevents phagosomal escape of *L*. *monocytogenes* in macrophages and creates conditions that support bacterial replication inside spacious vacuoles, which we termed rSLAPs. Our data show that rSLAPs (i) are single-membrane vacuoles with close spatial proximity to host cell mitochondria; ii) occur in the presence of PrfA inhibition and low expression of bacterial LLO, with LLO being required for vacuole formation and/or support of intravacuolar *L*. *monocytogenes* replication; iii) are not decorated by LAMP1 and LC3 and exhibit neutral pH and no cathepsin D at the time of extensive intravacuolar bacterial replication. Thus, rSLAPs display a combination of features previously described for SLAPs in macrophages, as well as eSLAPs and LisCVs in non-phagocytic cells (Table 1). The partial overlap of characteristics may indicate that spacious *Listeria* replication vacuoles are initiated through a common mechanism and that their fate is shaped by a combination of bacterial and host-derived factors. Importantly, the main feature distinguishing rSLAPs from these vacuolar compartments is progressive elimination of *L*. *monocytogenes* from infected macrophages by lysosomal delivery accompanied by macrophage survival. Thus, intravacuolar residence of *L*. *monocytogenes* in macrophages is not inevitably linked to intracellular bacterial persistence.

**Table 1:** Characteristics of *L*. *monocytogenes* intravacuolar niches.

	rSLAPs	SLAPs [18]	eSLAPs [22]	LisCVs [23]
host cell	macrophages	macrophages	epithelial cells	hepatocytes, trophoblast cells
Events upon initial host cell entry				
vacuole arising from	entry vacuole	entry vacuole	entry vacuole	bacterial entry after cytosolic transition
vacuole membrane	single membrane	single membrane	single membrane	single membrane
Intracellular replication niche				
host cell markers of replication niche		LC3, LAMP1, ATPase	LC3, LAMP1, Rab7	LAMP1, cathepsin D
	neutral pH	neutral pH	neutral pH	acidic pH
	close proximity to mitochondria			close proximity to mitochondria
bacterial factors required	low PrfA low LLO	low LLO	low LLO	ActA
Transformation of replication niche				
antimicrobial defense	progressive acquisition of lysosomal features (LAMP1, cathepsin D acidic pH)	non-degradative	non-degradative	lysosomal features (LAMP1, cathepsin D, acidic pH)
bacterial fate	elimination	intra-vacuolar persistence	release into cytoplasm upon vacuole rupture	intra-vacuolar persistence

Formation of rSLAPs, like SLAPs [18], occurred in the presence of low LLO expression. Extensive intravacuolar bacterial replication in rSLAPs required LLO, similar to what has been observed for eSLAPs in epithelial cells [22]. Yet, what role LLO plays in promoting rSLAP formation and intravacuolar replication of *L*. *monocytogenes* remains to be determined, specifically whether LLO concentration or hemolytic activity are dispensable, as has been reported for eSLAPs [22]. Our data suggest that rSLAPs progressively mature into degradative lysosomal compartments that aid elimination of *L*. *monocytogenes* from infected macrophages. This process may be triggered by accumulating LLO, increasingly compromising the integrity of the vacuolar membrane. Additional mechanisms driven by other virulence factors affected by PrfA inhibition, (e.g. PlcA and PlcB) might have a role to play. Future analyses of the *L*. *monocytogenes* secretome using fluorescence-tagging [22] should help delineate the spatio-temporal dynamics of *L*. *monocytogenes* virulence factor interactions with the different intracellular replication niches.

Activation of PrfA is tightly regulated by temperature, osmolarity, redox and metabolic cues that direct expression of *L*. *monocytogenes* virulence factors, while minimizing the fitness costs associated with PrfA activity [24]. We show that small molecule binding in the PrfA coactivator site leads to structurally similar positioning of the HTH motifs in the PrfA:KSK67 and PrfA:IWP-2 co-crystals (Fig 1G), which in case of the former is accompanied by impaired binding of PrfA to DNA [15]. Our data establish that small-molecule occupation of the PrfA coactivator binding site prevents vacuolar escape for *L*. *monocytogenes* and facilitates formation of rSLAPs, indicating a requirement for PrfA coactivation in the regulation of virulence genes that facilitate vacuolar escape. This interpretation aligns with observations in our study (Figs 1A–1D, 2C and 3E, and S1A Fig) and other reports [14,15] that small molecule-mediated inhibition of PrfA coactivation significantly impairs expression of the PrfA-controlled virulence genes *hly*, *plcA* and *plcB*. Nevertheless, it has been proposed that PrfA coactivation is dispensable for vacuolar escape based on findings that *L*. *monocytogenes* mutants featuring single amino acid substitutions in the PrfA coactivator site translocated into the cytoplasm despite significantly reduced *plcA* expression [8]. One possible explanation to reconcile the observations made with small molecule occupation versus genetic manipulation of the PrfA coactivator binding site is that a threshold expression of virulence factors is required for vacuolar escape (e.g. *hly*), which is not met in the context of inhibitory occupation of the PrfA coactivator site. In addition, the host cell environment might provide relevant context, as *L*. *monocytogenes* with a constitutively active PrfA replicated in eSLAPs in epithelial cells [22] but did not induce rSLAPs in macrophages.

Slow replication of intravacuolar *L*. *monocytogenes* has been hypothesized to enable asymptomatic carriage and chronic infection [17]. Intravacuolar *L*. *monocytogenes* were observed in liver macrophages of severe combined immunodeficiency (SCID) mice, which exhibit a protracted chronic infection [33]. But *L*. *monocytogenes* has also been found inside large, single-membrane vacuoles in splenic macrophages of immune-competent mice [34], suggesting that at least a subset of bacteria might assume an intravacuolar state, be it temporary or prolonged, within an immune-competent host. This scenario seems conceivable given the heterogeneity of intracellular bacterial fate upon infection [22] and recent insights that PrfA activity is regulated by peptides derived from nutritional sources as well as free fatty acids [14,35]. Nevertheless, whether *Listeria*-residence vacuoles *in vivo* resemble any of the vacuolar compartments defined in *in vitro* studies remains to be determined.

Our discovery, together with additional recent milestones in the understanding of molecular and structural requirements for PrfA functions [9,10,14,15] may be utilized to inform structure-activity-relationship-guided design of PrfA inhibitors that allow for selective targeting of DNA binding versus coactivation to define regulatory mechanisms underlying PrfA functions in different host environments. This will not only refine understanding of the temporal and spatial dynamics that enable *L*. *monocytogenes* to successfully infect the host but might also inspire the design of PrfA-targeted adjunct therapies to improve outcomes for patients with complications arising from listeriosis.

## Materials and methods

### Ethics statement

All procedures involving animals adhered to the National Health and Medical Research Council Australian Code for the Care and Use of Animals for Scientific Purposes and were approved by an Animal Ethics Committees of The University of Queensland (UQDI/571/12; UQDI/554/15, UQDI/059/19) and Monash University (MMCB/2016/16BC).

### Mice

Male and female C57BL/6 mice aged 8–14 weeks were bred in house under specific pathogen-free conditions or purchased from Animal Resources Centre (Perth, Australia). *Atg7*^fl/fl^ LysMCre^+/-^ and *Atg7*^wt/wt^ LysMCre^+/-^ mice were described previously [36]. Mice received food and water *ad libitum*.

### Primary cells and cell lines

Bone marrow was isolated from the femurs and tibias of mice. Bone marrow cells were differentiated for 6 days at 37°C and 5% CO_2_ in DMEM containing 20% L929-conditioned medium, 10% FBS, 2 mM L-glutamine, 1 mM sodium pyruvate, and 10 mM HEPES. Adherent cells were washed with warm PBS and collected with ice-cold EDTA (1 mM) in PBS. Cells were washed with ice-cold PBS and centrifuged at 244 x *g* for 5 min at 4°C. Cell pellets were resuspended in DMEM containing 10% L929-conditioned medium, 10% FBS, 2 mM L-glutamine, 1 mM sodium pyruvate, and 10 mM HEPES. Cells were seeded into multi-well tissue culture plates and incubated overnight at 37°C and 5% CO_2_ prior to experimentation. Immortalised bone marrow-derived macrophages (kindly provided by Eicke Latz, University of Bonn, Germany) were grown in DMEM containing Glutamax and 10% FBS, maintained at 37°C and 5% CO_2_. RAW264.7 macrophages, human monocytic THP-1 cells, and human colon carcinoma Caco-2 cells (HTB-37; kindly provided by Michael McGuckin, Mater Research Institute Brisbane, Australia) were maintained in complete DMEM (10% FBS, 1% L-glutamine, 1% sodium pyruvate, 1% HEPES). THP-1 cells were differentiated for 48 hours with 50 ng/mL of phorbol 12-myristate 13-acetate (PMA) prior to infection.

### Bacteria

*Listeria monocytogenes* strains 10403S (wild type), Δ*hly* [37], Δ*actA* [38], and GFP-10403S[39] (kindly provided by Eric Pamer, Memorial Sloan Kettering Cancer Centre, New York, USA); EGD-e [40], Δ*prfA* [41], PrfA^G145S^ [15]. GFP-expressing Δ*hly Listeria monocytogenes* was generated by transforming pAD_1_-cGFP [42] (kindly provided by Pascale Cossart; Institute Pasteur, France) into *L*. *monocytogenes* Δ*hly* [37]. Bacterial suspensions were streaked for single colonies onto Brain-Heart Infusion (BHI) agar and incubated for 24 h at 37°C. A single colony was used to inoculate 5 mL of BHI broth and incubated overnight at 37°C at 250 rpm. The bacterial culture was diluted 1/100 in BHI broth and incubated at 37°C at 250 rpm until early log-phase OD_600_ = 0.05–0.1. Bacteria were centrifuged for 10 min at 3270 x *g* at 4°C and bacteria resuspended in pre-warmed cell culture medium for infection assays. Bacteria were plated on BHI agar plates and colonies counted after 24 h of incubation at 37°C to confirm the colony forming units (CFU) in bacterial inocula.

*Legionella pneumophila* 130b Δ*flaA* [43] was plated on Buffered Charcoal Yeast Extract (BCYE) agar plates. Inoculum for *in vitro* infection assays was generated by collecting colonies in PBS and adjusting the bacterial numbers via UV-spectroscopy. To determine CFU, inocula were plated on BCYE agar plates and colonies were counted after 24–48 h of incubation at 37°C.

*Salmonella enterica* serovar Typhimurium (SL1344) was grown in LB broth at 37°C at 250 rpm to OD_600_ 0.6–0.8. Bacteria were centrifuged for 10 min at 3270 x *g* and 4°C, supernatant removed, and bacteria resuspended in pre-warmed cell culture medium for *in vitro* infection assays. Bacteria were plated on LB agar plates and colonies counted after 24–48 h of incubation at 37°C to confirm the CFU in bacterial inocula.

*Burkholderia thailandensis* strain E264 (ATCC; Cat# 700388) was grown overnight in Tryptic Soy Broth (TSB). The bacterial cultures were washed, diluted in PBS and resuspended in pre-warmed cell culture medium for *in vitro* infection assays. Bacteria were plated on LB agar plates and colonies counted after 48 h of incubation at 37°C to confirm the CFU in bacterial inocula.

*Coxiella burnetii* Nine Mile Phase II strain RSA439 was cultured for 7 days in liquid ACCM-2 at 37°C in 5% CO_2_ and 2.5% O_2_, washed and resuspended in pre-warmed cell culture medium for *in vitro* infection assays.

*Shigella flexneri* 2a strains (provided by Roy Robbins-Browne, University of Melbourne, Australia) were grown overnight in LB broth at 37°C in a shaker with aeration. Bacterial cultures were diluted 1:50 and grown to log phase for 2 h at 37°C in a shaking incubator. Bacteria were washed and diluted in cell culture medium. Bacteria were plated on LB agar plates and colonies counted after 24–48 h of incubation at 37°C to confirm the CFU in bacterial inocula.

*Pseudomonas aeruginosa* strain PAO1 was grown in LB broth at 37°C at 250 rpm to OD_600_ 0.6–0.8. Bacteria were washed and resuspended in pre-warmed cell culture medium. Bacteria were plated on LB agar plates and colonies counted after 24 h of incubation at 37°C to confirm the CFU in bacterial inocula.

*Staphylococcus aureus* strain NRS384 (BEI Resources; Cat# NR-46070) was grown overnight in BHI broth. Bacteria were washed and resuspended in pre-warmed cell culture medium. Bacteria were plated on LB agar plates and colonies counted after 24 h of incubation at 37°C to confirm the CFU in bacterial inocula.

### *L*. *monocytogenes* infection

IWP-2 or an equivalent volume of DMSO solvent control were added to BMM, RAW264.7 cells, THP-1 cells (all 1.5 x 10^5 in 48 well plates), or Caco-2 cells (2 x 10^5 per well in 24 well plates) 16 h prior to infection with *L*. *monocytogenes* (BMM, Raw264.7, THP-1—MOI 5; Caco-2 –MOI 20). Alternatively, inhibitors (IWP-2, LGK-974 [Tocris Bioscience, Active Biochem], KSK67 [S1 Methods], Oligomycin [Cayman Chemical], FCCP [Sigma-Aldrich], 2DG [Sigma-Aldrich], SS-31 [S1 Methods]) and solvent controls were added to BMM 1 h prior to infection with *L*. *monocytogenes*. For siRNA-mediated knock-down, BMM (0.5 x 10^6^ cells per well in 24 well plates) were transfected using Lipofectamine RNAiMAX (Life Technologies) and stealth siRNA targeting mouse *Porcn* (MSS225410/ Cat # 1320001, MSS 225411, Cat # 1320001, MSS225412, Cat # 1320001) or scrambled control siRNA (Cat# AM4611) in OptiMEM (Life Technologies). At 4–5 h after transfection, cell supernatants were removed and replaced with DMEM containing 10% L929-conditioned medium, 10% FBS, 2 mM L-glutamine, 1 mM sodium pyruvate, 10 mM HEPES. Knock-down of mRNA expression was confirmed by qRT-PCR. Cells were infected with *L*. *monocytogenes* (MOI 3–5 for CFU analyses, MOI 3 for microscopy) and the cell culture plates centrifuged at 335 x *g* for 2 min at room temperature. Cells were then incubated at 37°C for 30 min. To remove extracellular bacteria, cells were then washed twice with warm DMEM containing 50 μg/mL gentamicin, washed once with warm cell culture medium, and then further cultured in medium containing 5 μg/mL gentamicin alongside the inhibitor or solvent control at the appropriate concentration for the remainder of the experiments. To assess protection of intracellular *L*. *monocytogenes* from penicillin, 60 μg/ml penicillin was added to cell cultures 2 h before wash and lysis for CFU determination. Cell supernatants were used for assessing lactate dehydrogenase release (CytoTox96 Non-Radioactive Cytotoxicity Assay, Promega) to determine cell viability. For assessment of intracellular bacterial burden, cells were washed twice with warm PBS, lysed in 0.1% Triton X100 in PBS and cell lysates serially diluted and plated on BHI agar plates. The number of colonies were counted after 24 h of incubation at 37°C and the CFU/mL calculated. The time required [min] for a 50% increase or decrease in CFU was calculated by dividing the CFU difference between two time points by the time passed.

### *L*. *monocytogenes in vitro* growth

Overnight cultures of *L*. *monocytogenes* were sub-cultured and grown at 37°C to OD_600_ 0.05–0.1. Cultures were diluted to OD_600_ 0.01 in BHI and divided across individual culture tubes for each treatment. Small molecule inhibitors or equivalent volumes of DMSO as solvent control were added and bacteria incubated in a shaking incubator at 37°C. Absorbance at 600 nm was monitored over time.

### *Listeria monocytogenes* mRNA and protein expression

Overnight cultures of *L*. *monocytogenes* were sub-cultured in 5 mL BHI medium and grown at 37°C in a shaking incubator to OD_600_ 0.2. Inhibitors were added to individual culture tubes and the bacteria further incubated at 37°C. At each timepoint, the bacteria were centrifuged at 3270 x *g* and 4°C for 10 min and the supernatant discarded. For determination of bacterial gene expression, the bacterial pellet resuspended in lysozyme (2 mg/mL), incubated at 37°C for 2 h, then TRIzol (ThermoFisher Scientific) was added to each sample and RNA extracted. cDNA synthesis was performed with total RNA using GoScript cDNA synthesis kit with random hexamers (Promega). Quantitative PCR was performed with the Sybr Green PCR master mix (ThermoFisher Scientific) using ViiA7 (Applied Biosystems). Primers (Integrated DNA Technologies) are listed in S1 Table. Relative gene expression was calculated as 2^(Ct value 16S rRNA—Ct value gene of interest)^. For analyses of LLO protein expression, pellets were resuspended in 0.06 M Tris/HCl pH 6.8 with 2% SDS, 10% glycerol, 5% 2-mercaptoethanol, 0.05% bromophenol blue and incubated at 95°C for 5 min. Samples were separated by SDS-PAGE and transferred to nitrocellulose membranes. The membranes were blocked with 5% skim milk in TBS/0.1% Tween 20. Membranes were incubated overnight at 4°C with antibodies against LLO (1:1000; Abcam; host species: rabbit) and *Listeria* (1:2000; KPL Antibodies and Conjugates; host species: goat) diluted in 2.5% BSA in TBS with 0.1% Tween 20, followed by incubation with HRP-conjugated antibodies (1:10,000; goat anti-rabbit IgG: Cell Signaling Technology; donkey anti-goat IgG: Abcam) diluted in 5% skim milk powder in TBS with 0.1% Tween 20. SuperSignal West Dura extended duration substrate (ThermoFisher Scientific) was used to visualise the proteins, which were then imaged (Fusion SL, Vilber Lourmat) and analysed (ImageJ).

To assess *L*. *monocytogenes* gene and protein expression in infected cells, 1x10^6 Raw264.7 cells were seeded into 6-well plates and were pre-incubated with IWP-2 and DMSO as above. Cells were infected with *L*. *monocytogenes* (MOI 1) and cell culture plates centrifuged at 335 x *g* for 2 min at room temperature. Cells were then incubated at 37°C for 30 min. To remove extracellular bacteria, cells were then washed twice with warm DMEM containing 50 μg/mL gentamicin, washed once with warm cell culture medium, and then further cultured in medium containing 5 μg/mL gentamicin alongside the inhibitor or solvent control at the appropriate concentration for the remainder of the experiment. At the indicated time points, supernatants were collected and cells lysed in 0.1% Triton X100. Cells and supernatant were centrifuged at 3270 x *g* and 4°C for 10 min. The supernatant was removed, and the pellet resuspended in PBS, then centrifuged again at 3270 x *g* at 4°C for 10 min. The supernatant was removed, and the pellet incubated in lysozyme (2 mg/mL) for 2 h at 37°C. For gene expression analyses, Trizol was added and RNA was extracted. For protein expression analyses, pellets were resuspended in 0.06 M Tris/HCl pH 6.8 with 2% SDS, 10% glycerol, 5% 2-mercaptoethanol, 0.05% bromophenol blue at a 1:1 ratio (v:v) and incubated at 95°C for 5 min. *Listeria* protein expression was assessed by western blot as outlined above.

### Immunofluorescence microscopy

Cells were seeded onto sterile glass 15 mm coverslips (ProSciTech) in 10 μM IWP-2 or DMSO-containing media 16 h prior to *L*. *monocytogenes* infection. Where indicated, LysoTracker Deep Red (Life Technologies) was added to the cell cultures (50 nM), prior to cell fixation, and incubated for 15–30 min at 37°C, 5% CO_2_. Cells were then washed once with 37°C PBS and immediately fixed with 4% paraformaldehyde (ProSciTech) for 15 min at room temperature. For cell surface staining, rat anti-CD11b antibody (M1/70; BD Pharmingen; 2 μg/mL) was used in non-permeabilised cells. Rabbit anti-Tomm20 (Abcam; EPR15581, 0.5 μg/mL) and rat anti-Lamp1 (1D4B BD Pharmingen; 5 μg/mL) antibodies were used to detect mitochondria and lysosomes, respectively, in permeabilized cells. For visualising LC3 (rabbit anti-LC3B; Sigma-Aldrich #L7543; 4 μg/mL;) and Cathepsin D (rabbit anti-CatD; Abcam; EPR3057Y; 0.825 μg/mL), cells were fixed with cold methanol for 5 min at -20°C. Because methanol fixation reduced the GFP signal of GFP-expressing *Listeria*, an anti-*Listeria* antibody was used (KPL Antibodies and Conjugates; Cat# 5310–0320; 10 μg/mL). For all conditions, fixed cells were blocked and, as required, permeabilised in PBS, 0.1% Triton X-100 and 5% bovine serum albumin for 1 h at room temperature. Coverslips were incubated with primary antibodies diluted in blocking buffer for 1 h at room temperature, followed by secondary antibodies, conjugated to Alexa Fluor 488 (donkey anti-goat IgG H&L; Abcam, ab150129), Alexa Fluor 594 (donkey anti-rabbit IgG H&L, Abcam; ab150076; donkey anti-rat IgG H&L; ab150156) or Alexa Fluor 647 (donkey anti-rabbit IgG H&L; Abcam; ab150075) diluted in blocking buffer for 1 h at room temperature. Staining of uninfected cells as well as cells stained with secondary antibody only were assessed as controls in each experiment (S6 Fig). Nuclei were stained using DAPI (1 μg/mL; ThermoFisher Scientific) for 5 min at room temperature. Actin was visualized using Alexa Fluor 647 Phalloidin (200 nM; ThermoFisher Scientific) for 15 min at room temperature. Coverslips were mounted with ProLong Gold Antifade reagent (Invitrogen). Image acquisition was performed using an inverted and fully motorised Nikon/Spectral Spinning Disc Confocal microscope (X-1 Yokogawa spinning disc with Borealis modification) with a 100x NA 1.49 or 60x NA 1.49 Plan Apochromat oil immersion objective lens (Nikon). For lower magnification imaging, a 10x Plan Apochromat DIC N1 NA 0.5 or a 20x S Plan Fluor ELWD NA 0.5 objective lens (Nikon) were used. All images were acquired using a coupled device (CCD) camera (Andor Clara) and the Nikon elements imaging software (Nikon, Version 4.40). Each image was acquired through the entire cell every 0.5 μm. Image and colocalization analyses were carried out using the Nikon NIS-Elements AR Analysis software (Version 4.51.00). In summary, the background from all images was subtracted and the total area of *L*. *monocytogenes* per cell was determined. Subsequently, the area of *L*. *monocytogenes* colocalization with each co-stained cellular marker (LC3, LAMP1, LysoTracker, Cathepsin D or F-actin) per cell was determined in a blinded fashion. The percentage of colocalization over the total area of *L*. *monocytogenes* per cell was plotted using GraphPad Prism (Version 9.1.1). Maximum projection images were assembled using the FIJI (NIH) imaging software then copied to Adobe Illustrator (Version 24.3).

### Transmission electron microscopy

BMMs were seeded in 3.5 cm tissue culture dishes (Corning Inc.) at 0.1 x10^6^ cells/mL and incubated with 10 μM IWP-2 or DMSO overnight. Cells were infected with *L*. *monocytogenes* at MOI 3 and centrifuged at 335 x *g* for 2 min at room temperature. Infected cells were incubated at 37°C and 5% CO_2_ for 4 h, 10 h and 24 h, and then fixed with 2.5% glutaraldehyde (ProSciTech) in 0.1 M cacodylate buffer (ProSciTech), pH 7.4 for 10 min at room temperature. After fixation, the cells were washed with 0.1 M cacodylate buffer and post-fixed with 1% osmium tetroxide (ProSciTech)/1.5% potassium ferrocyanide (Sigma-Aldrich). The cells were then stained with 2% uranyl acetate for 1 h and dehydrated using ethanol. Cells were infiltrated with LX112 resin (ProSciTech), and resin filled gelatin capsules were inverted onto the dish and polymerised overnight at 60°C. The snapped-off capsules were sectioned at 90 nm on a Reichert Ultramicrotome, then stained with 5% uranyl acetate in 50% methanol and Reynold’s Lead and viewed on a JEOL 1011 electron microscope (JEOL Australasia) at 80 kV, and images were captured using the iTEM analysis program (Soft Imaging System, Olympus).

### *In vitro Legionella pneumophila* infection

iBMMs were seeded at 1x10^5^ cells/well in 24-well plates, and pre-incubated with 10 μM IWP-2 or DMSO for 16 h. Cells were infected with *L*. *pneumophila* at MOI 1 and centrifuged at 162 x *g* for 5 min at room temperature. Cells were incubated at 37°C for 2 h, the media was aspirated and replaced with media containing 100 μg/mL gentamicin and either DMSO or IWP-2, as appropriate. Cells were incubated 37°C for a further 1 h, and then washed three times with warm PBS. Media containing either DMSO or IWP-2 was replaced, and the cells incubated for the appropriate timepoints. Cells were lysed at 3 h, 24 h and 48 h by removing media and adding 0.05% digitonin in PBS and incubation for 5 min at RT. Cell lysates were serially diluted and plated on BCYE agar plates. Colonies were counted after 24–48 h of incubation at 37°C and the CFU/mL calculated.

### *In vitro Salmonella* Typhimurium infection

BMMs were seeded at 1.5 x 10^5 cells/well in 48 well plates and pre-incubated with 10 μM IWP-2 or DMSO for 16 h at 37°C. Cell were infected with *S*. Typhimurium (MOI 10). At 0.5 h post-infection, cells were washed twice with 50 μg/mL gentamicin in warm DMEM and twice with warm PBS. Media was replaced with media containing with 5 μg/mL gentamicin, and IWP-2 or DMSO as appropriate. At 0.5 h, 4 h and 24 h post-infection, cells were washed with PBS then lysed with 0.1% Triton X-100 in PBS after which cell lysates were serially diluted and plated onto LB agar plates. Colonies were counted after 24–48 h of incubation at 37°C and the CFU/mL calculated.

### *In vitro Burkholderia thailandensis* infection

iBMMs were seeded at 1 x 10^5 cells/well in 48 well plates and pre-incubated with 10 μM IWP-2 or DMSO for 16 h at 37°C. Cells were infected with *B*. *thailandensis* at MOI 1 and centrifuged at 500 x *g* for 2 min at room temperature. At 0.5 h post-infection, cells were washed twice with warm PBS, then incubated for 30 min with media supplemented with 250 μg/mL gentamicin to kill extracellular bacteria. The cells were then provided fresh media containing DMSO or IWP-2, and 50 μg/mL gentamicin. At 0.5 h, 4 h, 8 h and 24 h post-infection, cells were washed with PBS and lysed with 0.1% Triton X-100 in PBS, serially diluted and plated on LB agar plates. Colonies were counted after 48 h of incubation at 37°C and the CFU/mL calculated.

### *In vitro Coxiella burnetii* infection

THP-1 cells were seeded at 5 x 10^5 cells/well in 24 well plates and pre-incubated with 10 μM IWP-2 or DMSO for 24 h at 37°C. The bacteria were resuspended in pre-warmed culture medium and added to cells at MOI of 25. After 4 h, cells were washed once with PBS and fresh medium was added. Cells were lysed in dH_2_O at this timepoint (4 h = day 0) and at days 1, 3 and 5 post-infection. To quantify *C*. *burnetii*, Quant-iT PicoGreen dsDNA assay kit (ThermoFisher Scientific) was used. Genomic DNA was extracted from cells using the Zymo Quick-DNA Miniprep kit (Zymo Research), and qPCR was performed with primers detecting *ompA*. A fold difference in genomic equivalents over time was calculated relative to day 0.

### *In vitro Shigella flexneri* infection

iBMMs were seeded at 1x10^5^ cells/well in a 24-well plate and incubated with 10 μM IWP-2 or DMSO as solvent control for 16 h. Cells were infected with *S*. *flexneri* at MOI 100 and centrifuged at 554 x *g* for 5 min at room temperature before cells were incubated at 37°C for 1 h to allow for phagocytosis of bacteria. Gentamicin (100 μg/ ml) with either IPW2 or DMSO was then added to the monolayers and incubated at 37°C, 5% CO2 for either 1 h or 5 h to kill any extracellular bacteria. Following this, monolayers were washed 3 times with PBS to remove any gentamicin. Cells were lysed with 0.05% (w/v) digitonin in PBS for 5 min at RT at 2 h and 6 h post-infection. Serially diluted cell lysates were plated on LB agar plates, colonies counted after 24–48 h of incubation and the CFU/mL calculated.

### *In vitro Pseudomonas aeruginosa* infection

THP-1 cells were seeded at 1.5 x 10^5 cells/well in 48 well plates and pre-incubated with 10 μM IWP-2 or DMSO as solvent control for 16 h. Cells were infected with *P*. *aeruginosa* at MOI 5 and centrifuged at 335 x *g* for 2 min at room temperature. At 0.5 h post-infection, extracellular bacteria were removed by washing twice with 50 μg/mL gentamicin in warm DMEM and twice with warm PBS. Media containing DMSO or IWP-2, and 5 μg/mL gentamicin was replaced. At 0.5 h and 4 h post-infection, cells were washed with PBS and lysed in 0.1% Triton X-100 in PBS. Serially diluted cell lysates were plated on LB agar plates, colonies counted after 24 h of incubation at 37°C and CFU/mL calculated.

### *In vitro Staphylococcus aureus* infection

iBMMs were seeded at 1 x 10^5 cells/well in 48 well plates and pre-incubated with 10 μM IWP-2 or DMSO for 16 h. Cells were infected with *S*. *aureus* at MOI 5 and centrifuged at 500 x *g* for 2 min at room temperature. At 0.5 h post-infection, cells were washed twice with warm PBS and then incubated for 30 min at 37°C with media supplemented with 50 μg/mL gentamicin to kill extracellular bacteria. The media was then replaced with media containing either DMSO or IWP-2 and 50 μg/mL gentamicin. At 0.5 h, 4 h, 8 h and 24 h post-infection, cells were washed with PBS and lysed with 0.1% Triton X-100 in PBS. Serially diluted cell lysates were plated on BHI agar plates, colonies counted after 24 h of incubation at 37°C and CFU/mL calculated.

### PrfA purification and crystallization

Purification of PrfA for crystallization was performed as previously described [16]. Purified PrfA, in 200 mM NaCl and 20 mM NaP buffer at pH 6.5, was concentrated using Amicon Ultra centrifugal filter devices (Millipore) before being flash-cooled in liquid N_2_ and stored at -80°C. Prior to the crystallization setup, IWP-2 and DTT were added to the protein solution to final concentrations of 1 mM and 3.7 mM, respectively. Droplets of 1 μL protein solution at 3.1 mg/mL were mixed with 1 μL reservoir solution consisting of 29% (w/v) PEG 4000, 100 mM sodium citrate pH 5.5, and 18% (v/v) isopropanol. The dimethyl sulfoxide (DMSO) concentration was kept at 10% (v/v). Prior to vitrification, crystals were equilibrated for 2 days in a solution containing 35% (w/v) PEG 4000, 100 mM sodium citrate pH 5.5 and 1 mM IWP-2.

The synchrotron diffraction data at 100 K were collected at beamline P13 operated by EMBL Hamburg at the PETRA III storage ring (DESY, Hamburg, Germany). Diffraction images were processed with XDS [44] and scaled and merged using AIMLESS from the CCP4 software suite [45]. The structure was determined by molecular replacement with the program PHASER from the PHENIX program suite [46,47] using PrfA:KSK67 complex structure, PDB ID 6eut [16] as the search model. The atomic models were manually built using COOT [48] and refined with PHENIX Refine [49]. POLDER maps [50] were calculated with PHENIX. Data collection and refinement statistics are shown in S1 Table. Figures were prepared with CCP4mg [51]. The atomic coordinates and the structure factors have been deposited in the Protein Data Bank [52] (PDB ID 6T5I for PrfA:IWP-2).

### Molecular modelling

The molecular docking of IWP-2 and LGK-974 compounds was performed using the crystal structures of PrfA (PDB ID: 6EV0 and PDB ID: 5F1R) with the original ligands removed. These ligands are positioned at the A_I_, B_I_ and A_II_ sites in the 6EV0 structure, and at the A_I_ and B_II_ sites in the 5F1R structure. All the docking calculations were performed using the Glide extra precision (XP) mode in Schrödinger suite (Schrödinger, LLC, New York, NY, 2019). Initially, the protein structures were prepared for docking using the protein preparation wizard followed by energy minimization. The PrfA structure 6EV0 was used for docking calculations at A_I_, B_I_ and A_II_ sites, and the structure 5F1R was used for the B_II_ site. The receptor grid was generated using the position of inhibitors co-crystallized with the protein, into which Glide docks the compounds. All compounds were sketched in 3D format using the build module of Maestro and minimized through Macromodel to produce the lowest-energy conformation. Compounds were considered flexible, but the protein was kept rigid during docking calculations. The top-ranked pose in the calculation was considered as the most likely binding mode of the compound. A more detailed account of the docking studies is outlined in the S1 Methods.

### Statistical analyses

GraphPad Prism (v9.2.0; GraphPad Software, Inc.) was used to plot data and perform statistical tests as indicated in figure legends. The statistical test and number of replicates for each data set are stated in the figure legends. P values <0.05 were considered statistically significant.

## Supporting information

S1 Fig IWP-2 but not LGK974, diminishes expression of PrfA-controlled genes. **(A)** Expression of PrfA-controlled *L*. *monocytogenes* virulence genes in cell lysates (CL) and culture supernatants (SN) of murine RAW264.7 macrophages infected for 0.5 and 1 h in the presence of the PrfA inhibitor IWP-2 (10 μM), or DMSO as solvent control. **(B)** Examples of *L*. *monocytogenes* genes not affected by IWP-2 treatment in infected RAW264.7 macrophages. Data are from 4–5 independent experiments; means +/- sem are indicated. Groups at each condition and time point were compared by Mann-Whitney test. **(C)** *L*. *monocytogenes* was grown in brain heart infusion broth at 37°C for the times indicated in the presence of LGK-974 (10 μM) or DMSO. These cultures were run in parallel with those depicted in Fig 1C and are compared to the same DMSO controls. Data are means +/- sem of six independent cultures analyzed across three independent experiments. **(D)** LGK-974 did not diminished *L*. *monocytogenes* LLO protein expression analyzed by western blot in bacterial lysates grown in brain heart infusion broth at 37°C for the times indicated. Untreated starting cultures (ctrl), DMSO treatment and LLO-deficient Δ*hly L*. *monocytogenes* served as controls. Data are representative of 3 independent experiments with similar results.
(TIFF)

S2 Fig Molecular modelling of IWP-2 and LGK-974 interactions with PrfA. **(A-C)** Self-docking experiments to validate the docking process. The ligand from the previously published crystal structure (15, 16) is shown in green, the docked ligand IWP-2 in grey. (A) A_I_ site of PrfA (PDBID: 6EV0); (B) A_II_ site of PrfA (PDBID: 6EV0); (C) B_II_ site of PrfA (PDBID: 5F1R). **(d)** Docking of IWP-2 (blue) and LGK-974 (grey) into the A_I_ binding site of the *L*. *monocytogenes* PrfA homodimer (PDB ID: 6EV0). The phenyl substituent of IWP-2 is placed into the S1 pocket; LGK-974 has no substituent to occupy the S1 pocket. **(E)** Calculated docking scores for IWP-2 and LGK-974 at the A_I_ and B_I_ sites. The docking score for KSK67 at the A_I_ site is -9.9 kcal/mol (see S1 Methods for details) **(F)** Docking of IWP-2 and LGK-974 at the A_II_ and B_II_ site of the PrfA homodimer.
(TIFF)

S3 Fig IWP-2 (0.1, 1, 10 μM) dose-dependently diminished intracellular *L*. *monocytogenes* burden at 2–4 h post-infection of **(A)** murine RAW264.7 and human THP1 macrophage-like cells, and **(B)** human Caco-2 epithelial cells. D = DMSO solvent control. Data points represent 3 independent experiments, each performed in technical triplicates; means +/- sem are indicated. Two-way ANOVA with Dunnett multiple comparison correction. *p<0.05, **p<0.01, ***p<0.001, ****p<0.0001 **(C)** Addition of IWP-2 (0.1, 1, 10 μM) to murine bone marrow-derived macrophages 0.5 h after initial infection with *L*. *monocytogenes* dose-dependently diminished intracellular bacterial burden at 4 h post-infection but did not exhibit cytotoxic effects on murine bone marrow-derived macrophages as determined by LDH release assay. D = DMSO solvent control. Bacterial burden data are means +/- sem of 3 independent experiments each performed in technical triplicates. One-way ANOVA with Dunnett multiple comparison correction. *p<0.05; LDH release data are means +/- sd of triplicates of one representative experiment of 3 independent experiments. **(C)** IWP-2 (0.1, 1, 10 μM) did not impair *L*. *monocytogenes* replication in brain heart infusion broth at 37°C. Means +/- sem of 4 independent cultures. **(D)** Equivalent intracellular *L*. *monocytogenes* burden in murine bone marrow-derived macrophages transfected with PORCN-specific siRNA when compared to srcambled control RNA. Intracellular bacterial burden at 4 h post-infection normalized to bacterial uptake at 0.5 h post-infection. Data points represent 5 independent experiments, each performed in technical triplicates; means +/- sem are indicated. **(E)** Pre-incubation with LGK-974 (10 μM) did not diminished intracellular *L*. *monocytogenes* burden at 4 h post-infection (MOI 3) of murine bone marrow-derived macrophages (BMM), murine RAW264.7 macrophage-like cells, and human intestinal epithelial Caco-2 cells. D = DMSO solvent control. Data points represent 3–5 independent experiments, each performed in technical triplicates; means +/- sem are indicated. **(F)** The PrfA inhibitor IWP-2 does not impair macrophage control of bacterial pathogens that do not express PrfA. Macrophage cultures were infected with *Legionella pneumophila*, *Salmonella enterica* serovar Thyphimurium, *Burkholderia thailandensis*, *Coxiella burnetii*, *Shigella flexneri*, *Pseudomonas aeruginosa*, or *Staphylococcus aureus* in the presence of IWP-2 (10 μM) or DMSO as solvent control. Intracellular bacterial burden was determined by genome quantification (*C*. *burnetii*) or CFU determination (all other bacteria). Data points are from 2–3 independent experiments each performed in technical duplicates; means +/- sem for n = 3, means +/- range for n = 2. Two-way ANOVA with Sidak correction for multiple comparisons; *p<0.05, **p<0.01.
(TIFF)

S4 Fig Intracellular replication and elimination of *L*. *monocytogenes* upon PrfA inhibition occurs independent of ActA. Murine bone marrow-derived macrophages were infected with *L*. *monocytogenes* deficient for actin-assembly inducing protein (*ΔactA*) in the presence of IWP-2 (10 μM) or DMSO as solvent control. **(A)** Cell viability assessment by lactate dehydrogenease release (LDH). Means +/- sd of a representative experiment performed in triplicates. **(B)** Intracellular bacterial burden was assessed as colony forming units (CFU) at the times indicated. Data points are means +/- sem from 3 independent experiments, each performed in technical triplicates. (n.d. not determined due to extensive cell death).
(TIFF)

S5 Fig Interference with mitochondrial functions does not alter intracellular *L*. *monocytogenes* replication in spacious vacuoles or the cytoplasm. (A) Close proximity of intracellular GFP-expressing *L*. *monocytogenes* and mitochondria (visualized with anti-Tom20) in murine bone marrow-derived macrophages in the presence of DMSO or IWP-2 (10 μM) analyzed by confocal fluorescence microscopy (scale bars 10 μm; scale bars of insets 5 μm). Images are representative of similar results obtained in at least 3 independent experiments. **(B)** Close proximity of mitochondria and spacious vacuoles in IWP-2-treated bone marrow-derived macrophages as revealed by transmission electron microscopy (scale bar 1 μm). Similar observations were made in two independent experiments. **(C)** Murine bone marrow-derived macrophages were infected with *L*. *monocytogenes* in the presence of IWP-2 (10 μM) or DMSO as solvent control. Inhibitors of mitochondrial functions FCCP (10 μM), oligomycin (4 μM), 2-deoxy-2-glucose (200 μM) and SS-31 (10 μM) were added 1 h prior to infection. Intracellular bacterial burden was determined at the indicated time points. Data represent means +/- sem of 3–5 independent experiments each performed in triplicates.
(TIFF)

S6 Fig Fluorescence confocal microscopy controls. **(A)** Fluorescence confocal microscopy images of uninfected murine bone marrow-derived macrophages cultured in the presence of DMSO or IWP-2 (10 μM) and stained for LC3, LAMP1, cathepsin D, Tom20, Lysotracker, and DNA (DAPI). **(B)** Images of cells incubated with secondary antibody only as specificity controls for each of the markers. Images are representative of at least three independent experiments.
(TIFF)

S1 Table Crystallography data collection and refinement statistics. (DOCX)

S2 Table Primer sequences. (DOCX)

S1 Methods Supplemental methods. (DOCX)
