# Supplementary figures and images for: Inhibition of the master regulator of Listeria monocytogenes virulence enables bacterial clearance from spacious replication vacuoles in infected macrophages
Source: PLoS Pathog. 2022 Jan 10;18(1):e1010166. doi: 10.1371/journal.ppat.1010166 (PMC8746789; doi:10.1371/journal.ppat.1010166)

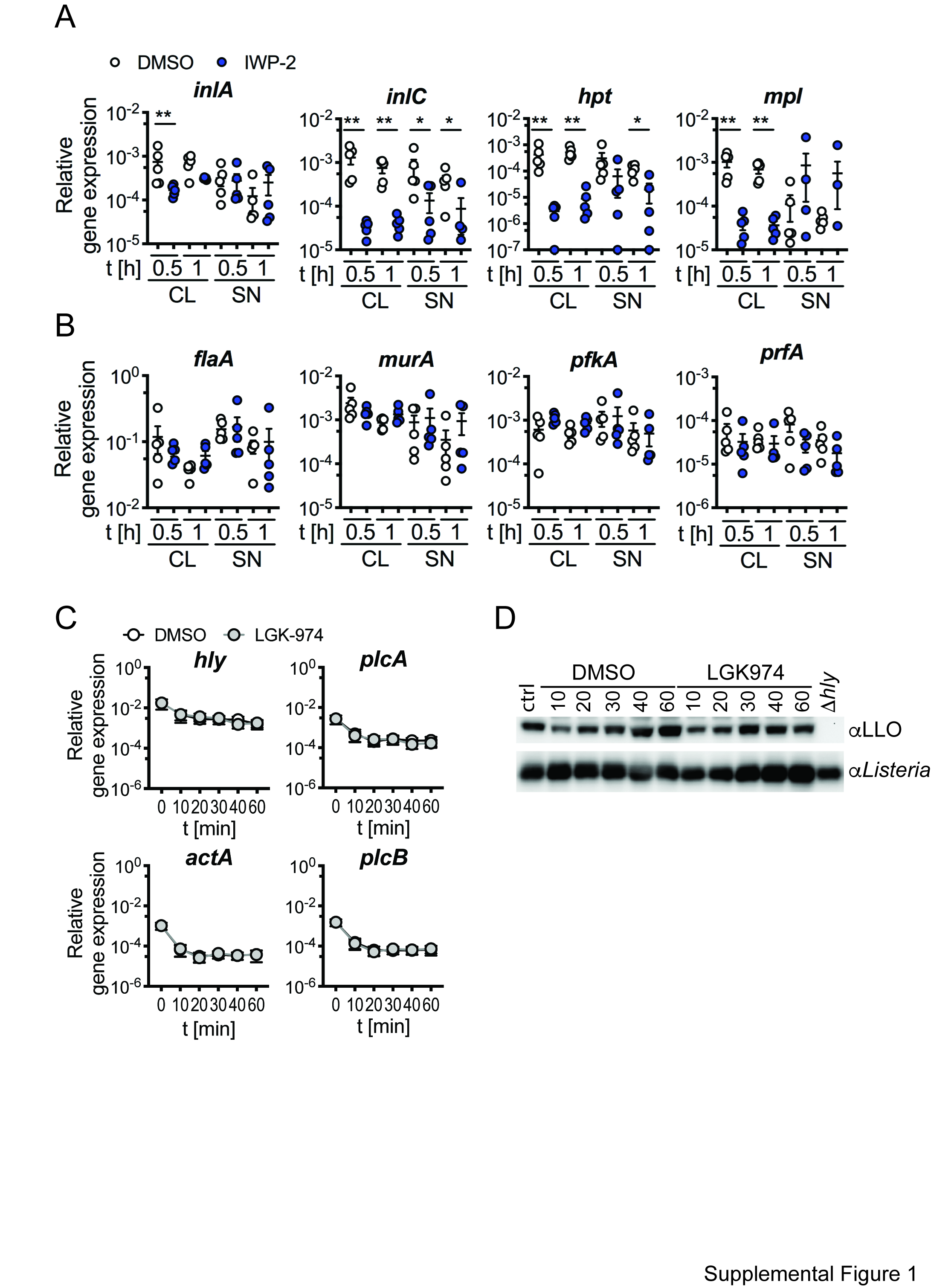

Supplement: S1 Fig — (A) Expression of PrfA-controlled L. monocytogenes virulence genes in cell lysates (CL) and culture supernatants (SN) of murine RAW264.7 macrophages infected for 0.5 and 1 h in the presence of the PrfA inhibitor IWP-2 (10 μM), or DMSO as solvent control. (B) Examples of L. monocytogenes genes not affected by IWP-2 treatment in infected RAW264.7 macrophages. Data are from 4–5 independent experiments; means +/- sem are indicated. Groups at each condition and time point were compared by Mann-Whitney test. (C) L. monocytogenes was grown in brain heart infusion broth at 37°C for the times indicated in the presence of LGK-974 (10 μM) or DMSO. These cultures were run in parallel with those depicted in Fig 1C and are compared to the same DMSO controls. Data are means +/- sem of six independent cultures analyzed across three independent experiments. (D) LGK-974 did not diminished L. monocytogenes LLO protein expression analyzed by western blot in bacterial lysates grown in brain heart infusion broth at 37°C for the times indicated. Untreated starting cultures (ctrl), DMSO treatment and LLO-deficient Δhly L. monocytogenes served as controls. Data are representative of 3 independent experiments with similar results. (TIFF) [file ppat.1010166.s001.tiff]

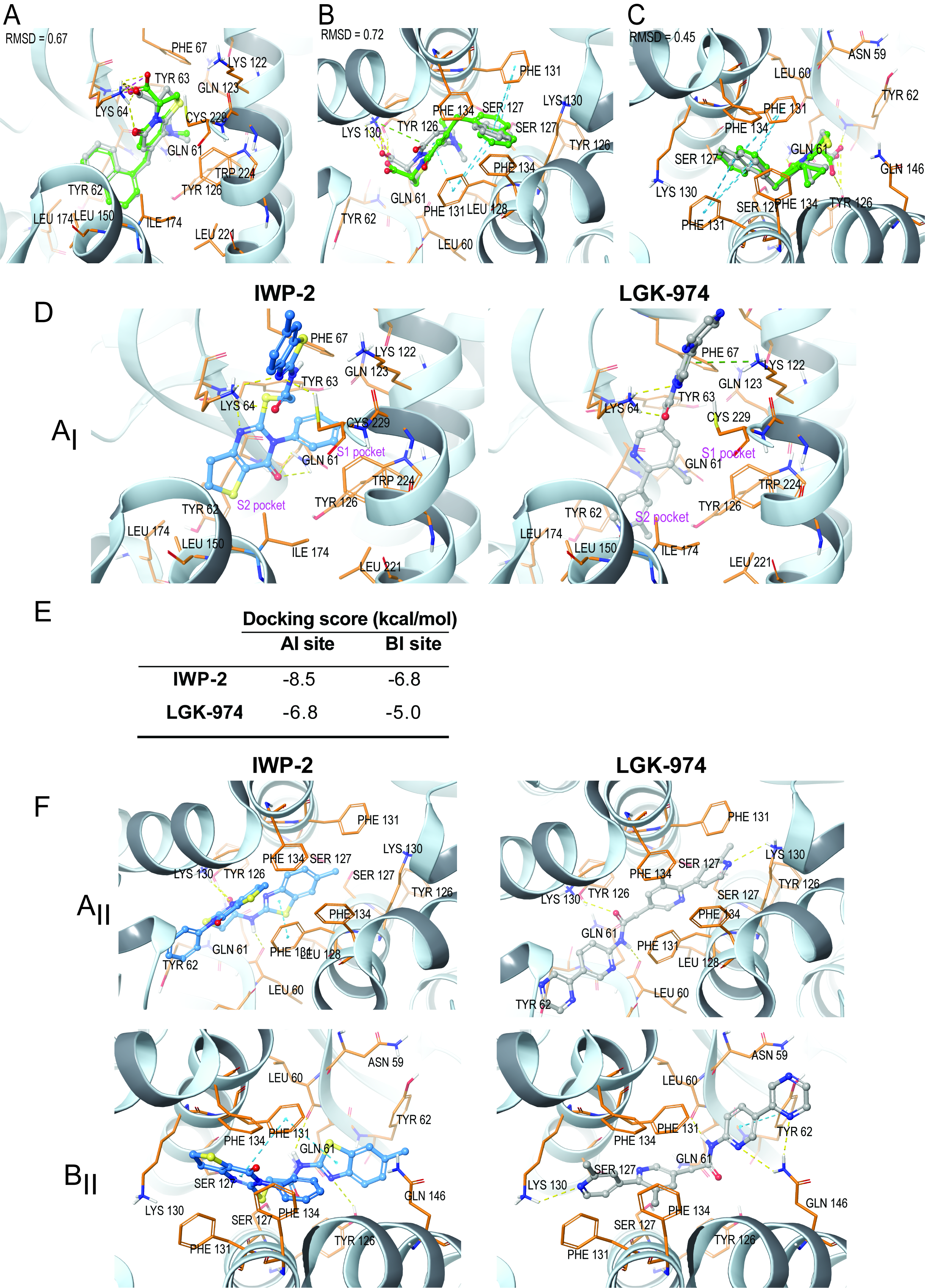

Supplement: S2 Fig — (A-C) Self-docking experiments to validate the docking process. The ligand from the previously published crystal structure (15, 16) is shown in green, the docked ligand IWP-2 in grey. (A) AI site of PrfA (PDBID: 6EV0); (B) AII site of PrfA (PDBID: 6EV0); (C) BII site of PrfA (PDBID: 5F1R). (d) Docking of IWP-2 (blue) and LGK-974 (grey) into the AI binding site of the L. monocytogenes PrfA homodimer (PDB ID: 6EV0). The phenyl substituent of IWP-2 is placed into the S1 pocket; LGK-974 has no substituent to occupy the S1 pocket. (E) Calculated docking scores for IWP-2 and LGK-974 at the AI and BI sites. The docking score for KSK67 at the AI site is -9.9 kcal/mol (see S1 Methods for details) (F) Docking of IWP-2 and LGK-974 at the AII and BII site of the PrfA homodimer. (TIFF) [file ppat.1010166.s002.tiff]

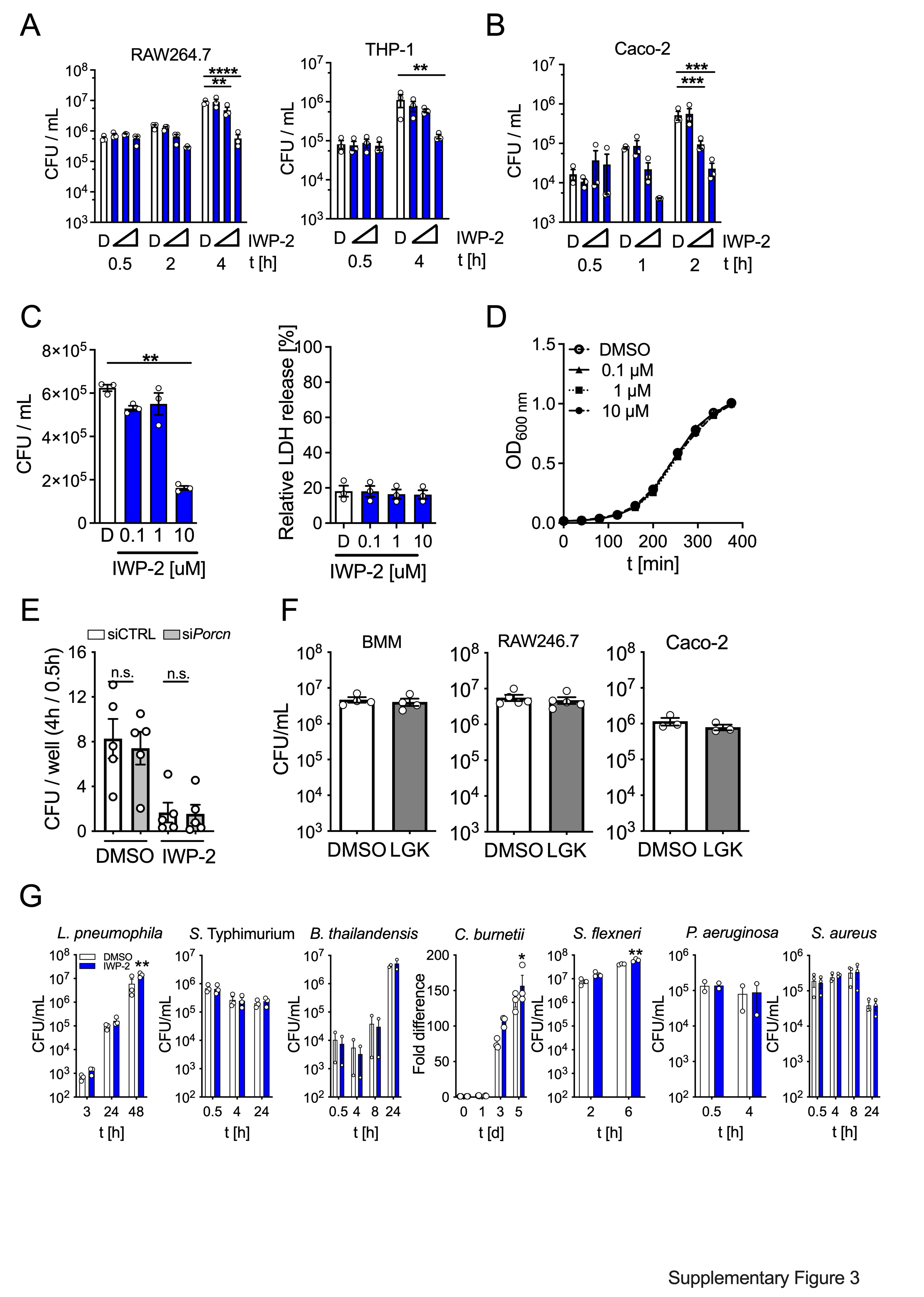

Supplement: S3 Fig — IWP-2 (0.1, 1, 10 μM) dose-dependently diminished intracellular L. monocytogenes burden at 2–4 h post-infection of (A) murine RAW264.7 and human THP1 macrophage-like cells, and (B) human Caco-2 epithelial cells. D = DMSO solvent control. Data points represent 3 independent experiments, each performed in technical triplicates; means +/- sem are indicated. Two-way ANOVA with Dunnett multiple comparison correction. *p<0.05, **p<0.01, ***p<0.001, ****p<0.0001 (C) Addition of IWP-2 (0.1, 1, 10 μM) to murine bone marrow-derived macrophages 0.5 h after initial infection with L. monocytogenes dose-dependently diminished intracellular bacterial burden at 4 h post-infection but did not exhibit cytotoxic effects on murine bone marrow-derived macrophages as determined by LDH release assay. D = DMSO solvent control. Bacterial burden data are means +/- sem of 3 independent experiments each performed in technical triplicates. One-way ANOVA with Dunnett multiple comparison correction. *p<0.05; LDH release data are means +/- sd of triplicates of one representative experiment of 3 independent experiments. (C) IWP-2 (0.1, 1, 10 μM) did not impair L. monocytogenes replication in brain heart infusion broth at 37°C. Means +/- sem of 4 independent cultures. (D) Equivalent intracellular L. monocytogenes burden in murine bone marrow-derived macrophages transfected with PORCN-specific siRNA when compared to srcambled control RNA. Intracellular bacterial burden at 4 h post-infection normalized to bacterial uptake at 0.5 h post-infection. Data points represent 5 independent experiments, each performed in technical triplicates; means +/- sem are indicated. (E) Pre-incubation with LGK-974 (10 μM) did not diminished intracellular L. monocytogenes burden at 4 h post-infection (MOI 3) of murine bone marrow-derived macrophages (BMM), murine RAW264.7 macrophage-like cells, and human intestinal epithelial Caco-2 cells. D = DMSO solvent control. Data points represent 3–5 independent experiments, each p [file ppat.1010166.s003.tiff]

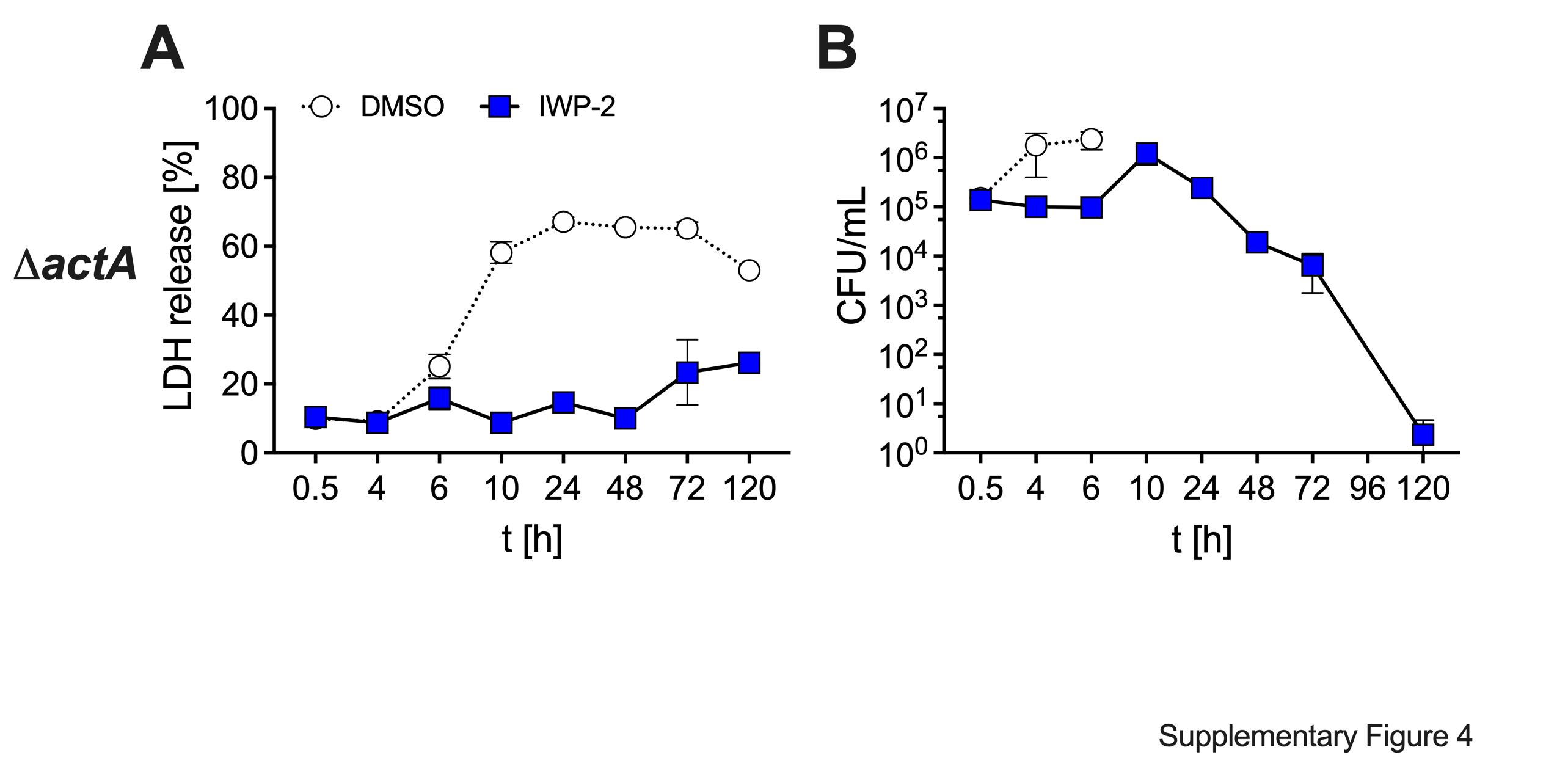

Supplement: S4 Fig — Murine bone marrow-derived macrophages were infected with L. monocytogenes deficient for actin-assembly inducing protein (ΔactA) in the presence of IWP-2 (10 μM) or DMSO as solvent control. (A) Cell viability assessment by lactate dehydrogenease release (LDH). Means +/- sd of a representative experiment performed in triplicates. (B) Intracellular bacterial burden was assessed as colony forming units (CFU) at the times indicated. Data points are means +/- sem from 3 independent experiments, each performed in technical triplicates. (n.d. not determined due to extensive cell death). (TIFF) [file ppat.1010166.s004.tiff]

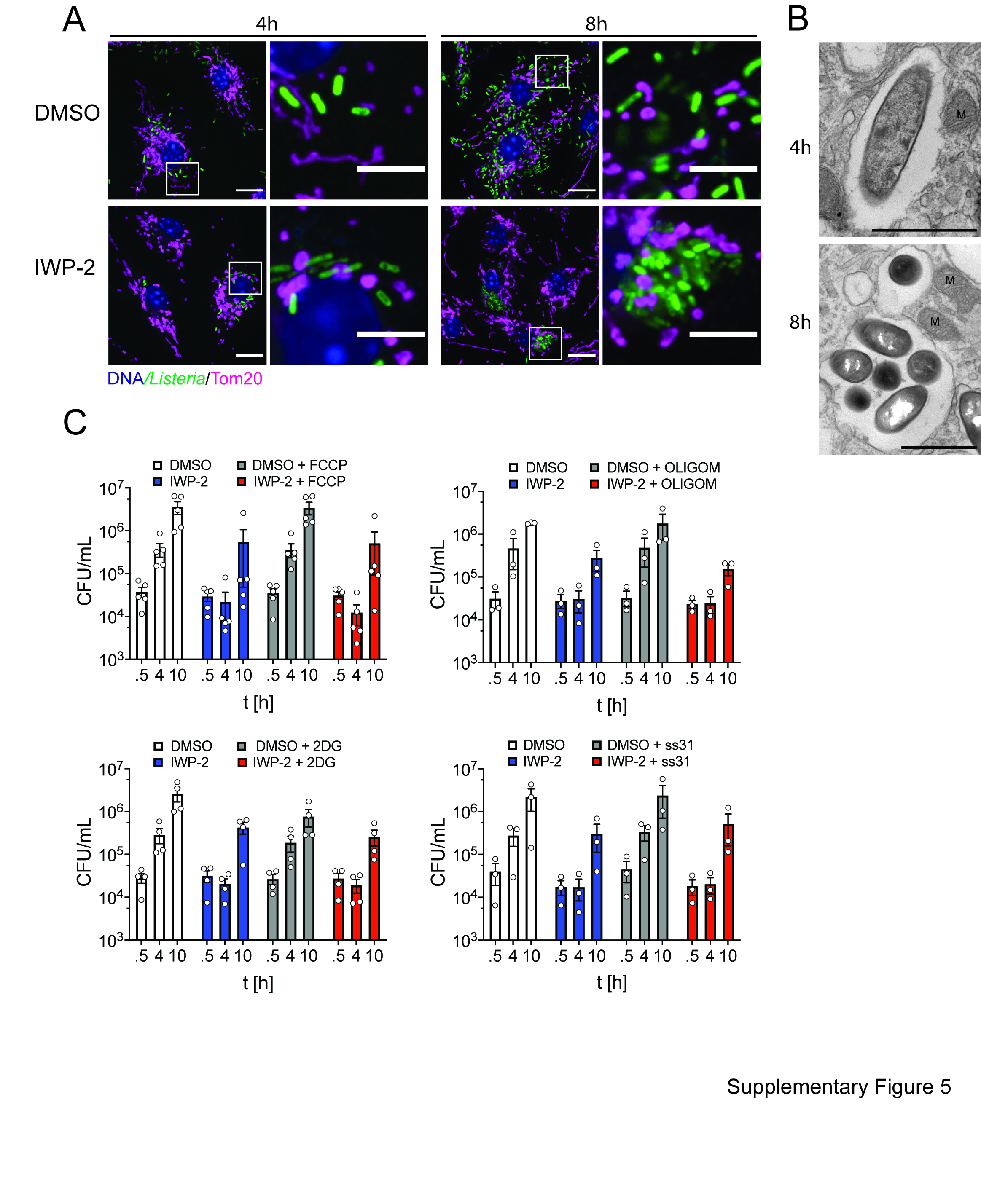

Supplement: S5 Fig — (A) Close proximity of intracellular GFP-expressing L. monocytogenes and mitochondria (visualized with anti-Tom20) in murine bone marrow-derived macrophages in the presence of DMSO or IWP-2 (10 μM) analyzed by confocal fluorescence microscopy (scale bars 10 μm; scale bars of insets 5 μm). Images are representative of similar results obtained in at least 3 independent experiments. (B) Close proximity of mitochondria and spacious vacuoles in IWP-2-treated bone marrow-derived macrophages as revealed by transmission electron microscopy (scale bar 1 μm). Similar observations were made in two independent experiments. (C) Murine bone marrow-derived macrophages were infected with L. monocytogenes in the presence of IWP-2 (10 μM) or DMSO as solvent control. Inhibitors of mitochondrial functions FCCP (10 μM), oligomycin (4 μM), 2-deoxy-2-glucose (200 μM) and SS-31 (10 μM) were added 1 h prior to infection. Intracellular bacterial burden was determined at the indicated time points. Data represent means +/- sem of 3–5 independent experiments each performed in triplicates. (TIFF) [file ppat.1010166.s005.tiff]

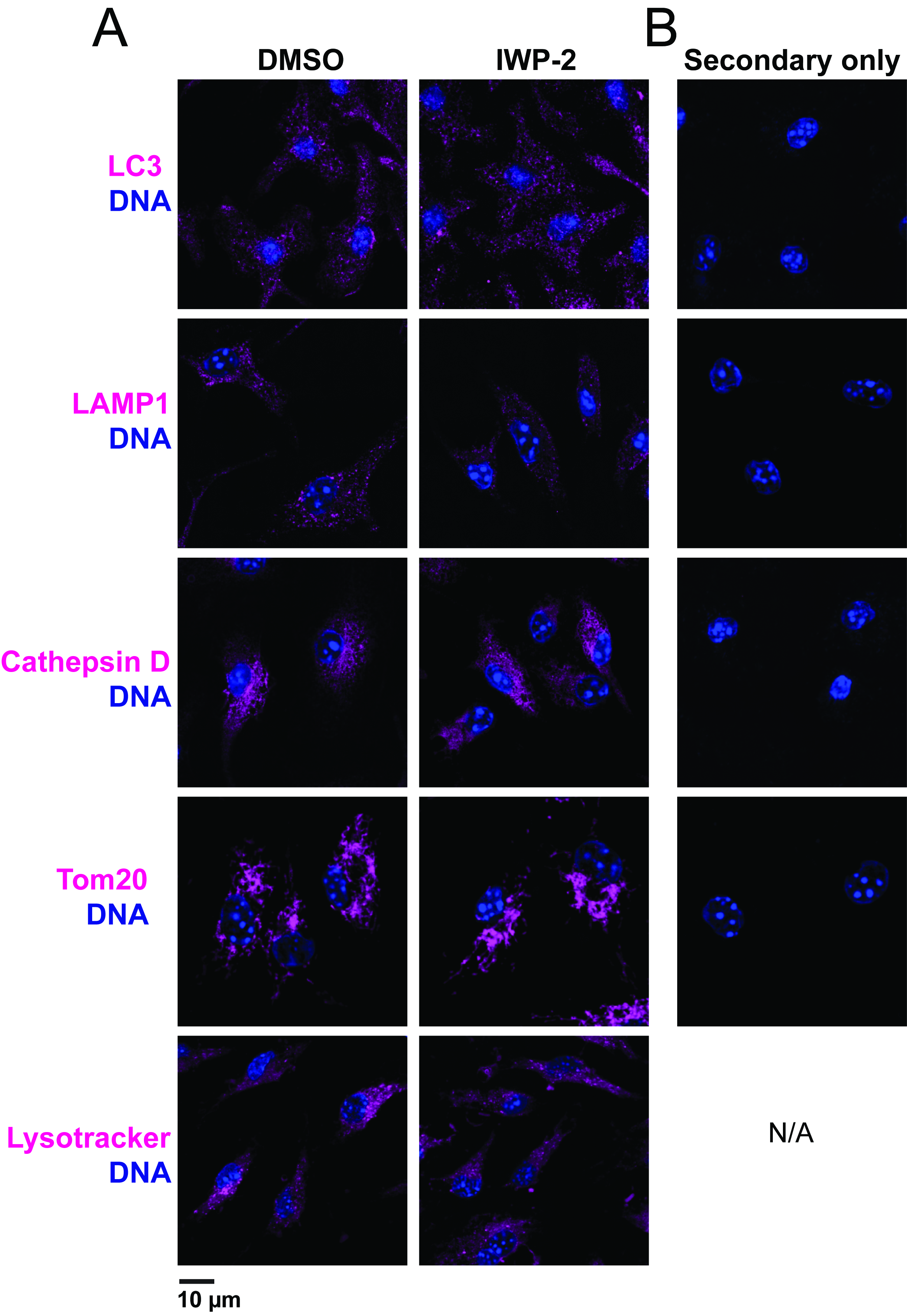

Supplement: S6 Fig — (A) Fluorescence confocal microscopy images of uninfected murine bone marrow-derived macrophages cultured in the presence of DMSO or IWP-2 (10 μM) and stained for LC3, LAMP1, cathepsin D, Tom20, Lysotracker, and DNA (DAPI). (B) Images of cells incubated with secondary antibody only as specificity controls for each of the markers. Images are representative of at least three independent experiments. (TIFF) [file ppat.1010166.s006.tiff]
