# Supplementary material for: Inhibition of the master regulator of Listeria monocytogenes virulence enables bacterial clearance from spacious replication vacuoles in infected macrophages
Source: PLoS Pathog. 2022 Jan 10;18(1):e1010166. doi: 10.1371/journal.ppat.1010166 (PMC8746789; doi:10.1371/journal.ppat.1010166)
Supplement: S1 Table — (DOCX) [file ppat.1010166.s007.docx]

**Supporting Information**

**S1 Table. Crystallography data collection and refinement statistics**

|  | **PrfA_WT_-IWP2** |
| --- | --- |
| **Data collection** |  |
| Space group | P2_1_2_1_2_1_ |
| Cell dimensions |  |
| *a*, *b*, *c* (Å) | 47.95, 92.67, 100.45 |
| α, β, γ (°) | 90, 90, 90 |
| Resolution (Å)* | 46.3-2.0 (2.07-2.0) |
| *R*_merge_ | 0.135 (2.078) |
| *R*_PIM_ | 0.040 (0.609) |
| *I* / σ*I* | 12.6 (1.2) |
| Completeness (%) | 99.2 (98.8) |
| Redundancy | 12.9 (13.1) |
| CC1/2 | 0.999 (0.435) |
|  |  |
| **Refinement** |  |
| No. unique reflections | 30998 (3033) |
| *R*_work_ | 0.205 (0.252) |
| *R*_free_ | 0.246 (0.294) |
| No. atoms |  |
| Protein | 3793 |
| IWP2 | 31 |
| DMSO, Na | 48, 2 |
| Waters | 101 |
| *B*-factors (Å^2^) |  |
| Protein | 52.2 |
| IWP2 | 68.0 |
| DMSO, Na | 58.9, 47.2 |
| Water | 47.4 |
| R.m.s. deviations |  |
| Bond lengths (Å) | 0.009 |
| Bond angles (°) | 0.96 |
| Ramachandran |  |
| Favored (%) | 97.8 |
| Outliers (%) | 0.0 |
| PDB code | 6T5I |

Diffraction data were collected on a single crystal. *Values in parentheses are for the highest resolution shell. Resolution limits were determined by applying a cut-off based on the mean intensity correlation coefficient of half-datasets, CC_1/2_.
