# Supplementary material for: Inhibition of the master regulator of Listeria monocytogenes virulence enables bacterial clearance from spacious replication vacuoles in infected macrophages
Source: PLoS Pathog. 2022 Jan 10;18(1):e1010166. doi: 10.1371/journal.ppat.1010166 (PMC8746789; doi:10.1371/journal.ppat.1010166)
Supplement: S2 Table — (DOCX) [file ppat.1010166.s008.docx]

**Supporting Information**

**S2 Table. Primer sequences**

| Species | Gene | Forward (5’-3’) | Reverse (5’-3’) | Reference |
| --- | --- | --- | --- | --- |
| 1 | *actA* | GATTTATGCGTGCGATGATGG | ACCTCGCTTGGTTGCTCTTC | this study |
| 1 | *flaA* | AGTAAGCATCCAAGCGTCTG | ACCAGCAACTGTAGAACCAC | this study |
| 1 | *hly* | gcaatttcgagcctaaccta | actgcgttgttaacgtttga | [1] |
| 1 | *hpt* | GGACGCCAAAATTGAAAAGAGG | ACCCGGAACGATAAACATTCC | this study |
| 1 | *inlA* | ACCTTTAGCCAACCTGTCAC | GTTTCTTTGCCGTCCACATG | this study |
| 1 | *inlB* | AGGCATCTACAAACTTCCACG | TTTCGGGCTTCTCTATCAACAG | this study |
| 1 | *inlC* | GTCTGTAACACTTTGCTTCCC | GACCAACGCCTATTAACCAAG | this study |
| 1 | *mpl* | CGGGATTGGAATACGAAGGG | TTCTTATTCGCCCATCTCGC | this study |
| 1 | *murA* | GAACCAATTGCACATCCACC | AGTAACAGTTGATGCGACAGG | this study |
| 1 | *pfkA* | CAAGTACACGGTCAAATGCAG | AGCTGAAGGCGTAATGTCTG | this study |
| 1 | *plcA* | TACTCCCAGAACTGACACGAG | GAATTACTTGGTTAGGTGCGC | this study |
| 1 | *plcB* | CCCTCCAGGCTACCACTGTG | GTAGTCCGCTTTCGCCCTT | this study |
| 1 | *prfA* | caatgggatccacaagaata | agcctgctcgctaatgactt | [1] |
| 1 | 16S rRNA | TTAGCTAGTTGGTAGGGT | AATCCGGACAACGCTTGC | [2] |
| 2 | *ompA* | CAGAGCCGGGAGTCAAGCT | CTGAGTAGGAGATTTGAATCGC | [3] |

1 *Listeria monocytogenes*; 2 *Coxiella burnetii*

**References**

1. Klein PG, Juneja VK. Sensitive detection of viable Listeria monocytogenes by reverse transcription-PCR. Appl Environ Microbiol. 1997;63(11):4441-8.

2. Fraser KR, Sue D, Wiedmann M, Boor K, O'Byrne CP. Role of sigmaB in regulating the compatible solute uptake systems of Listeria monocytogenes: osmotic induction of opuC is sigmaB dependent. Appl Environ Microbiol. 2003;69(4):2015-22.

3. Jaton K, Peter O, Raoult D, Tissot JD, Greub G. Development of a high throughput PCR to detect Coxiella burnetii and its application in a diagnostic laboratory over a 7-year period. New Microbes New Infect. 2013;1(1):6-12.
