# Supplementary material for: Inhibition of the master regulator of Listeria monocytogenes virulence enables bacterial clearance from spacious replication vacuoles in infected macrophages
Source: PLoS Pathog. 2022 Jan 10;18(1):e1010166. doi: 10.1371/journal.ppat.1010166 (PMC8746789; doi:10.1371/journal.ppat.1010166)
Supplement: S1 Methods — (DOCX) [file ppat.1010166.s009.docx]

**Supporting Information**

Supplementary Methods

Synthesis of KSK67

*Chemicals*

Anhydrous dichloromethane was purchased from Sigma-Aldrich, then stored and dispensed from an Innovative Technology PureSolv solvent purification system. Anhydrous ethanol was prepared by refluxing with magnesium and catalytic iodine, and then distilling the mixture under argon. Dry acetyl chloride was prepared by refluxing with phosphorus pentachloride under argon, then distilling the mixture under argon. All other chemicals were used as purchased without further purification.

*Purification and analysis*

Preparative HPLC mobile phases were defined as follows: solvent A was MilliQ H_2_O with 0.1% trifluoroacetic acid, and solvent B was MeCN–H_2_O (90:10, v/v) with 0.1% trifluoroacetic acid. Analytical LCMS mobile phases were defined as follows: solvent A was MilliQ H_2_O with 0.1% formic acid, and solvent B was MeCN–H_2_O (90:10, v/v) with 0.1% formic acid. LCMS analysis was carried out on a Shimadzu LCMS-2020 system equipped with a Phenomenex Luna 3 micron C18(2) 150 × 2.00 mm column (P/No 00F-4251-BO) and a SPD-M20A diode array detector.

*Characterization*

^1^H and ^13^C NMR spectra were recorded on Bruker Avance 600 spectrometers at 298 K in deuterated solvents indicated and referenced to tetramethylsilane (^1^H/^13^C: *δ* 0.00/77.0 for CDCl_3_) or residual solvent signals (^1^H/^13^C: *δ* 2.50/39.52 for *d_6_*-DMSO).

*Compound synthesis*

Dry acyl chloride (AcCl, 880 μL, 12.5 mmol) and 2-cyclopropylacetonitrile (0.500 g, 6.17 mmol) were successively added dropwise to dry ethanol (6.0 mL) under argon in an ice bath. The reaction was warmed rapidly to room temperature, stirred for 16 h under argon, before drying *in vacuo* to give a waxy solid. To this, dry dichloromethane (9.0 mL), cysteine methyl ester hydrochloride (225 mg, 1.31 mmol), and then triethylamine (0.18 mL, 1.29 mmol) was added. A white precipitate formed within minutes of triethylamine addition. The same amount of cysteine methyl ester hydrochloride and triethylamine were again added 3 h later, then 70 h later the reaction was partitioned between dichloromethane and saturated sodium bicarbonate solution. The aqueous phase was extracted with dichloromethane. The combined organic phases were dried with magnesium sulfate, concentrated *in vacuo*, then purified by silica gel chromatography (25% ethyl acetate/petroleum spirit) to give the expected dihydrothiazole product as a colourless oil (67 mg, 0.34 mmol, 13 %). The ^1^H NMR spectrum (CDCl_3_) matched that reported in the literature [1].

The procedure by Good et al. was adapted as follows [2]. To a solution of Meldrum’s acid (526 mg, 3.65 mmol) and chloroacetic acid (288 mg, 2.55 mmol) in dry dichloromethane (11.0 mL), cooled in an ice bath, was added 4-dimethylaminopyridine (78 mg, 0.64 mmol) and then triethylamine (1.27 mL, 9.11 mmol). Propylphosphonic anhydride (3.60 mL, 50 % w/w solution in ethyl acetate, 6.05 mmol) was added dropwise to the mixture over 10 minutes, resulting in a yellow mixture. The reaction vessel was left in the ice bath and allowed to warm to room temperature overnight. After 48 h, the mixture was cooled in an ice bath, before carefully adding aqueous potassium hydrogen sulfate (3 % w/w, 10 mL). The mixture was extracted with dichloromethane (×3), and then the combined extracts were washed with aqueous potassium hydrogen sulfate (3 % w/w, 4 × 10 mL), brine, dried over magnesium sulfate, and concentrated *in vacuo*. The resulting reddish-brown solid, which was the expected product (540 mg, 2.45 mmol, 96 %), was used without purification. The ^1^H NMR spectrum (CDCl_3_) matched that reported in the literature [3].

The procedure by Good et al. was followed [4]. To a mixture of the methyl ester (31 mg, 0.16 mmol) and the Meldrum’s acid derivative (105 mg, 0.476 mmol) in dichloroethane (0.70 mL) was added trifluoroacetic acid (0.012 mL). The reaction was heated to 120 °C in a microwave reactor for 3 minutes, at which point LCMS indicated that the methyl ester starting material had been completely consumed. The mixture was diluted with dichloromethane, washed with saturated sodium bicarbonate solution, and the aqueous phase was extracted twice with dichloromethane. The combined organic phases were washed with brine, dried over magnesium sulfate, and then concentrated *in vacuo*. Purification by silica gel chromatography (15% to 100 % ethyl acetate/petroleum spirit) gave the desired product as a yellow oil (42 mg, 0.14 mmol, 88 %). [α^22^_D_] -170° (*c* = 1.0, CHCl_3_). The ^1^H NMR spectrum (CDCl_3_) matched that reported in the literature [3].

The procedure by Good et al. was followed [2]. The alkyl chloride starting material (42 mg, 0.14 mmol), the boronic acid (39 mg, 0.21 mmol), bis(triphenylphosphine)palladium chloride (10 mg, 0.014 mmol), and potassium fluoride (20 mg, 0.35 mmol) were dried briefly under high vacuum in a microwave vial equipped with a magnetic stir bar. After backfilling with argon, dry methanol (0.70 mL) was added. The vial was sealed and stirred at room temperature to dissolve most of the material. The mixture was then heated in a microwave reactor at 140°C for 10 minutes, at which point LCMS indicated complete consumption of the alkyl chloride starting material. Saturated sodium bicarbonate solution was added, and the mixture was extracted with ethyl acetate (×3). The combined extracts were washed with brine, dried over magnesium sulfate, and then concentrated *in vacuo*. The residue was purified by silica gel chromatography (30 to 100 % ethyl acetate/petroleum spirit) to give the expected adduct (37 mg, 0.091 mmol, 65 %). The ^1^H NMR spectrum (in CDCl_3_) was consistent with the structure, but did not match that reported in the literature [2,5]. We surmise that the NMR solvent had been incorrectly reported [2,5], as the spectrum of the isolated product (peaks listed below) was very similar to the ^1^H NMR spectrum (in CDCl_3_) for an analogue without the aromatic methyl group, as reported elsewhere [6].

^1^H NMR (CDCl_3_, 600 MHz): δ 8.03 (d, 1H, *J* = 8.3 Hz), 7.79 (d, 1H, *J* = 8.5 Hz), 7.51 (m, 1H), 7.46 (m, 1H), 7.25 (d, 1H, *J* = 7.1 Hz), 7.15 (d, 1H, *J* = 7.3 Hz), 5.72 (s, 1H), 5.55 (dd, 1H, *J* = 8.5, 2.2 Hz), 4.47 (d, 1H, 17.3 Hz), 4.31 (d, 1H, *J* = 17.3 Hz), 3.78 (s, 3H), 3.65 (dd, 1H, *J* = 11.8, 8.6 Hz), 3.49 (dd, 1H, *J* = 11.8, 2.1 Hz), 2.69 (s, 3H), 1.65 (m, 1H), 0.98 – 0.94 (m, 1H), 0.93 – 0.88 (m, 1H), 0.74 (m, 1H).

The procedures by Good et al. [2] and Chorell et al. [7] were adapted. Aqueous lithium hydroxide (1 M, 1.05 eq., 96 μL) was added dropwise to a vigorously stirred solution of the methyl ester (37 mg, 0.091 mmol) in tetrahydrofuran (2.7 mL) cooled in an ice bath. The reaction was kept in the ice bath and allowed to warm up to room temperature overnight. After 15 h, a white precipitate had formed, and LCMS indicated almost complete consumption of the starting material. The mixture was partitioned between dichloromethane and hydrochloric acid (1 M, aq). The aqueous phase, approximately pH 1, was extracted with dichloromethane. The combined extracts were dried over magnesium sulfate, and then concentrated *in vacuo* to give a white residue, which was triturated with diethyl ether before drying *in vacuo* to give a white solid (32 mg, 0.082 mmol, 90 %). The crude product was purified by preparative HPLC (50 to 100 % solvent B over 15 minutes) and then lyophilised to give the pure racemic product (15 mg, 0.038 mmol, 42 %) [α^22^_D_] 0° (*c* = 1.0, DMSO). The ^1^H NMR spectrum (*d*_6_-DMSO) matched that reported [2].

Synthesis of SS-31

*General experimental*

Analytical LC−MS analysis was conducted on a Shimadzu LCMS 2020 instrument. Reversed phase HPLC purification was conducted on an Agilent Preparative HPLC 1260 Infinity series instrument. Eluent 1: 0.05% formic acid in water (A) and 0.05% formic acid in acetonitrile (B). Eluent 2: 0.1% trifluoroacetic acid (TFA) in water (A) and 0.1% TFA in acetonitrile (B). HPLC column 1: Agilent Eclipse XDB phenyl, 3.0 × 100 mm, 3.5 μ. HPLC column 2: Agilent Eclipse XDB phenyl, 30 × 100 mm, 5 μ. HPLC *method 1*: eluent 1, column 1, flow 1 mL/min. Ratios refer to solvents A and B, respectively: 95:5 to 50:50, 5 min; 50:50 to 0:100, 0.5 min, 0:100, 1 min. HPLC *method 2*: eluent 2, column 2, flow 20 mL/min. Ratios refer to solvents A and B, respectively: 95:5 to 60:40, 8 min; 60:40 to 0:100, 1 min; 0:100, 2 min.

*Materials*

Tentagel SRAM Rink amide resin (SV 0.24 mmol/g) was purchased from Rapp Polymere. 1-Ethyl-3-(3-dimethylaminopropyl)carbodiimide (EDC), O-(1H-6-Chlorobenzotriazole-1-yl)-1,1,3,3-tetramethyluronium hexafluorophosphate (HCTU), and Fmoc-D-Arg(Pbf)-OH were purchased from Chem-Impex International Inc. (Wood Dale, IL, USA). Fmoc-L-Phe-OH and Fmoc-L-Lys(Boc)-OH were purchased from Iris Biotech. Fmoc-L-Tyr(2,6-DiMe)-OH was purchased from Key Organics. Peptide grade trifluoroacetic acid (TFA), piperidine, *N,N*-dimethylformamide (DMF) and *N*,*N*-diisopropyl amine (DIPEA) were purchased from AusPep (Melbourne, Australia). Dichloromethane (DCM) and methanol (MeOH) and diethyl ether were HPLC grade. Triisopropylsilane (TIS) was purchased from Sigma Aldrich and used as received.

Peptide synthesis was conducted using standard Fmoc SPPS protocols [8]. The starting Tentagel SRAM resin (3520 mg, 0.845 mmol, 0.24 mmol/g) was washed with DMF (×3) prior to deprotection. Fmoc deprotection was carried out with two successive treatments of the resin with piperidine in DMF (30% v/v, *ca*. 10 mL per gram of resin; 1 × 10 min, 1 × 20 min), followed by washing with DMF (×3). Fmoc-L-Phe-OH and Fmoc-L-Lys(Boc)-OH were sequentially coupled using HCTU/DIPEA in DMF as follows: a mixture of the amino acid (2.53 mmol, 3 equiv.) and HCTU (2.66 mmol, 3.1 equiv.) in DMF (10 mL) was treated with DIPEA (5.06 mmol, 6 equiv.) at room temperature. After 5 min, the pre-activated amino acid solution was added to the resin. The resin was gently agitated for 1 h, after which the solvent was drained, and the resin was washed with DMF (×3). Following the addition of Fmoc-L-Phe-OH, the Fmoc group was removed as above, and the coupling similarly continued for Fmoc-L-Lys(Boc)-OH. Thereafter, the coupling of Fmoc-L-Tyr(2,6-DiMe)-OH and Fmoc-D-Arg(Pbf)-OH was carried out using EDC/HOBt in DCM/DMF (10:1) as follows: a mixture of the amino acid (1.7 mmol, 2 equiv.) and HOBt (1.7 mmol, 2 equiv.) was suspended in a mixture of DCM/DMF (10:1, 9 mL). After cooling in an ice bath, EDC (1.7 mmol, 2 equiv.) was added in one portion. Cooling was maintained for 5 min, after which the resultant clear solution was added to the resin. After gentle agitation for 30 min, the solvent was drained, and the resin was washed with DMF (×3). Following the addition of Fmoc-L-Tyr(2,6-DiMe)-OH, the Fmoc group was removed as above, and the coupling similarly continued for Fmoc-D-Arg(Pbf)-OH. During the synthesis, coupling efficiency was monitored after each step using the Kaiser test [9]. Upon completion of the synthesis, the *N*-terminal Fmoc group was removed as above. The resin was successively washed with DMF, DCM and MeOH (×3 each), followed by drying under vacuum to give a final resin weight of 3.77 g. The peptide was cleaved from the resin (3.77 g) by treatment with TFA/TIS/H_2_O (95:2.5:2.5, 40 mL) for 1 h at room temperature. The cleavage solution was drained from the resin, collected and evaporated under reduced pressure. The crude residue was triturated in diethyl ether, and the resulting solid collected by vacuum filtration. The solid was washed with diethyl ether and dried under vacuum to yield crude product (368 mg). Purification by rp-HPLC (method 2) gave the trifluoroacetate salt of SS-31 as a white solid (294 mg, >97% purity), *t*_R_ 2.1 (method 1). (ES) m/z 640.5 (MH^+^), 300.7 (MH_2_^2+^). The overall yield of SS-31 from Tentagel SRAM resin was 35%.

Molecular Modelling

To first validate the docking process, self-docking experiments were performed by re-docking the ring-fused 2-pyridones co-crystallised with PrfA into their respective crystal structures with the ligands removed. The best docking pose was compared with the crystal pose through superposition. Nearly identical orientation and low root-mean-square deviation (rmsd) values were obtained (S2A-C Figs). The PrfA inhibitors based on the ring-fused 2-pyridone scaffold reported by Kulen et al. possess hydrophobic substituents that form hydrophobic interactions with the side-chains of the hydrophobic residues in two distinct binding pockets, S1 and S2, within the A_I_ and B_I_ binding sites [10]. Docking of IWP-2 into the A_I_ and B_I_ sites revealed a similar binding mode, with the ring-fused pyrimidone moiety of IWP-2 projected into the S2 pocket and the phenyl substituent occupying the S1 pocket (S2D Fig). In addition, the nitrogen of the benzothiazole ring and the carbonyl group of the peptide bond displayed hydrogen bond interactions with the amino group of lysine (Lys) 64 (S2D Fig), one of the key residues reported for PrfA activity [11]. Docking of LGK-974 predicted that its bipyridine moiety projects into the S2 pocket and forms hydrogen bond interactions with Lys 64, yet lacks a hydrophobic substituent and cannot reach the S1 pocket (S2D Fig). The docking scores based on all possible different orientations suggested that IWP-2 binds with high binding affinity at the A_I_ site (-8.5 kcal/mol), when compared to LGK-974 (-6.8 kcal/mol) (S2E Fig). The docking score for IWP-2 compared favourably with the best-scoring docking pose of the co-crystallized ring-fused 2-pyridone at the A_I_ site (-9.9 kcal/mol). These data supported the hypothesis that occupation of the S1 pocket may be responsible for the ability of IWP-2 to inhibit PrfA activity, compared to the inactive LGK-974, which does not occupy this pocket. Modelling of binding in the B_I_ site resulted in low scores for both IWP-2 and LGK-974 (IWP-2 -6.8 kcal/mol; LGK-974 -5.0 kcal/mol) (S2E Fig), highlighting that some side chains have to move from their positions in ligand-free PrfA to allow the 2-pyridones and the IWP-2 compound to bind. In particular, the sidechain of Lys130 is blocking part of the B_I_ binding site, possibly explaining the differences in docking scores.

The PrfA A_II_ and B_II_ sites are hydrophobic pockets and share a common region contributed by residues Phe131 and Phe134 from the αC helix of both PrfA monomers. Both IWP-2 and LGK-974 could be modelled into the A_II_ and B_II_ sites. At the A_II_ site, IWP-2 bound by positioning the benzothiazole group sandwiched between Phe131 and Phe134 residues, whereas the B_II_ site was occupied by the ring-fused pyrimidone moiety (S3F Fig). LGK-974 is a larger molecule with linear topology. The docking studies suggested that LGK-974 binds non-selectively at the A_II_ and B_II_ adjacent binding sites. The 2',3-dimethyl-2,4'-bipyridine moiety was predicted to be placed in between Phe131 and Phe134 residues at both A_II_ and B_II_ sites, with hydrogen bonds formed with the amino group of Lys130 from the adjacent subunit (S3F Fig). The calculated docking scores for IWP-2 and LGK-974 at the A_II_ and B_II_ sites were similar ranging between -8.9 to -7.6 kcal/mol and did not reflect patterns attributable to IWP-2 and LGK-974 differentially inhibiting PrfA-controlled *L. monocytogenes* virulence gene expression. Taken together, the docking studies supported the hypothesis that IWP-2 may directly inhibit PrfA functions by binding the A_I_ pocket of the PrfA homodimer.

**References**

1. Emtenas H, Taflin C, Almqvist F. Efficient microwave assisted synthesis of optically active bicyclic 2-pyridinones via delta2-thiazolines. Mol Divers. 2003;7(2-4):165-9. Epub 2004/02/12. PubMed PMID: 14870846.

2. Good JA, Andersson C, Hansen S, Wall J, Krishnan KS, Begum A, et al. Attenuating Listeria monocytogenes Virulence by Targeting the Regulatory Protein PrfA. Cell Chem Biol. 2016;23(3):404-14. Epub 2016/03/19. doi: 10.1016/j.chembiol.2016.02.013. PubMed PMID: 26991105; PubMed Central PMCID: PMCPMC4802734.

3. Sellstedt M, Prasad GK, Krishnan KS, Almqvist F. Directed diversity-oriented synthesis. Ring-fused 5-to 10-membered rings from a common peptidomimetic 2-pyridone precursor. Tetrahedron Lett. 2012;53(45):6022-4. doi: 10.1016/j.tetlet.2012.08.100. PubMed PMID: WOS:000310760800011.

4. Good JA, Silver J, Nunez-Otero C, Bahnan W, Krishnan KS, Salin O, et al. Thiazolino 2-Pyridone Amide Inhibitors of Chlamydia trachomatis Infectivity. J Med Chem. 2016;59(5):2094-108. Epub 2016/02/06. doi: 10.1021/acs.jmedchem.5b01759. PubMed PMID: 26849778.

5. ENGSTRÖM P KS, Chorell E, Bengtsson C, Good J, Almqvist F, Bergström S, inventorPreparation of thiazolopyridinone derivatives useful for treatment of Chlamydia infections.2014.

6. Emtenas H, Ahlin K, Pinkner JS, Hultgren SJ, Almqvist F. Design and parallel solid-phase synthesis of ring-fused 2-pyridinones that target pilus biogenesis in pathogenic bacteria. J Comb Chem. 2002;4(6):630-9. Epub 2002/11/12. PubMed PMID: 12425608.

7. Chorell E, Pinkner JS, Phan G, Edvinsson S, Buelens F, Remaut H, et al. Design and synthesis of C-2 substituted thiazolo and dihydrothiazolo ring-fused 2-pyridones: pilicides with increased antivirulence activity. J Med Chem. 2010;53(15):5690-5. Epub 2010/07/01. doi: 10.1021/jm100470t. PubMed PMID: 20586493; PubMed Central PMCID: PMCPMC2963145.

8. Jensen KJ, Shelton PT, Pedersen SL. Peptide Synthesis and Applications Second Edition Preface. Peptide Synthesis and Applications, 2nd Edition. 2013;1047:V-V. doi: Book_Doi 10.1007/978-1-62703-544-6. PubMed PMID: WOS:000325639900001.

9. Kaiser E, Colescott RL, Bossinger CD, Cook PI. Color test for detection of free terminal amino groups in the solid-phase synthesis of peptides. Anal Biochem. 1970;34(2):595-8. Epub 1970/04/01. doi: 10.1016/0003-2697(70)90146-6. PubMed PMID: 5443684.

10. Kulen M, Lindgren M, Hansen S, Cairns AG, Grundstrom C, Begum A, et al. Structure-Based Design of Inhibitors Targeting PrfA, the Master Virulence Regulator of Listeria monocytogenes. J Med Chem. 2018;61(9):4165-75. Epub 2018/04/19. doi: 10.1021/acs.jmedchem.8b00289. PubMed PMID: 29667825.

11. Xayarath B, Volz KW, Smart JI, Freitag NE. Probing the role of protein surface charge in the activation of PrfA, the central regulator of Listeria monocytogenes pathogenesis. PLoS One. 2011;6(8):e23502. Epub 2011/08/23. doi: 10.1371/journal.pone.0023502. PubMed PMID: 21858145; PubMed Central PMCID: PMCPMC3155570.
